# Toward the Rational Design of Molecular Field-Coupled Nanocomputing Candidates

**DOI:** 10.1021/acs.nanolett.6c02459

**Published:** 2026-09-12

**Authors:** Federico Ravera, Leonardo Medrano Sandonas, Andrea Vezzoli, Yuri Ardesi, Mariagrazia Graziano, Gianluca Piccinini, Gianaurelio Cuniberti

**Affiliations:** † Department of Electronics and Telecommunication, 19032 Politecnico di Torino, Torino 10129, Italy; ‡ Institute for Materials Science and Max Bergmann Center of Biomaterials, 9169 TUD Dresden University of Technology, Dresden 01062, Germany; ¶ Department of Chemistry, 4591 University of Liverpool, Liverpool L69 7ZD, United Kingdom; § Department of Applied Science and Technology, Politecnico di Torino, Torino 10129, Italy; ∥ Cluster of Excellence CARE, TUD Dresden University of Technology and RWTH Aachen, Dresden 01062, Germany; ⊥ Cluster of Excellence CeTI, 9169 TUD Dresden University of Technology, Dresden 01062, Germany

**Keywords:** Molecular nanocomputing, quantum chemistry, molecular design, device functionality

## Abstract

Molecular Field-Coupled Nanocomputing (MolFCN) is a promising beyond-CMOS paradigm in which information is propagated electrostatically rather than through charge transport, enabling ultra-low-power logic. However, identifying molecules with stable logic states and reliable information propagation remains challenging. Here, we introduce LUFFY (Layered Unified Framework for MolFCN systematic analYsis), a framework for the rational design and validation of MolFCN molecular candidates. Starting from 32 synthetically accessible molecules, LUFFY combines conformational sampling and electrostatic analysis in neutral and oxidized states to derive molecular response descriptors. We extract V_in_-to-Aggregated-Charge Transcharacteristics (VACTs), capturing the field-induced charge response, and develop energy-averaged models that account for conformational diversity and are validated against ab initio molecular dynamics. We further demonstrate stable information propagation at the device level. LUFFY establishes a unified, transferable, and scalable strategy linking molecular structure to circuit functionality, providing a foundation for data-driven molecular discovery for ultra-low-power computing.

## Graphical abstract

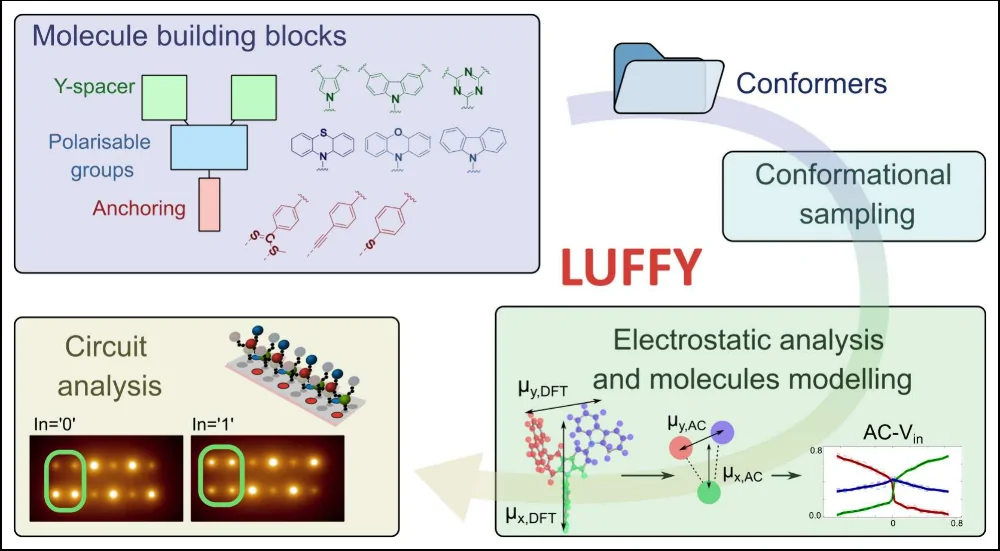

As CMOS-technology approaches its limits in power density and switching speed, currentless computing paradigms are being explored. Among these, Molecular Field-Coupled Nanocomputing (MolFCN) aims to drastically reduce energy dissipation in logic operations—as further CMOS scaling becomes increasingly constrained by thermal and switching bottlenecks.^1,2^ MolFCN—implementation of the Quantum-dot Cellular Automata (QCA)—encodes binary states through charge localization in molecular groups named dots (Figure 1(a)).^3^ MolFCN propagates logical states through electrostatic interactions.^2,4,5^ Moreover, vertical fields, named clock fields (*E*_*ck*_), enforce either the *Hold* (logic-stable) or *Null* (reset) configurations,^6^ enabling currentless computation, thus ultralow power dissipation. MolFCN remained a theoretical concept in recent decades; however, a concrete technological pathway to implementation has recently been introduced via a dielectric nano-trench architecture (Figure 1(b)).^7,8^ This approach represents a significant step in bridging the gap between theoretical frameworks and experimental realization.

**Figure 1:**
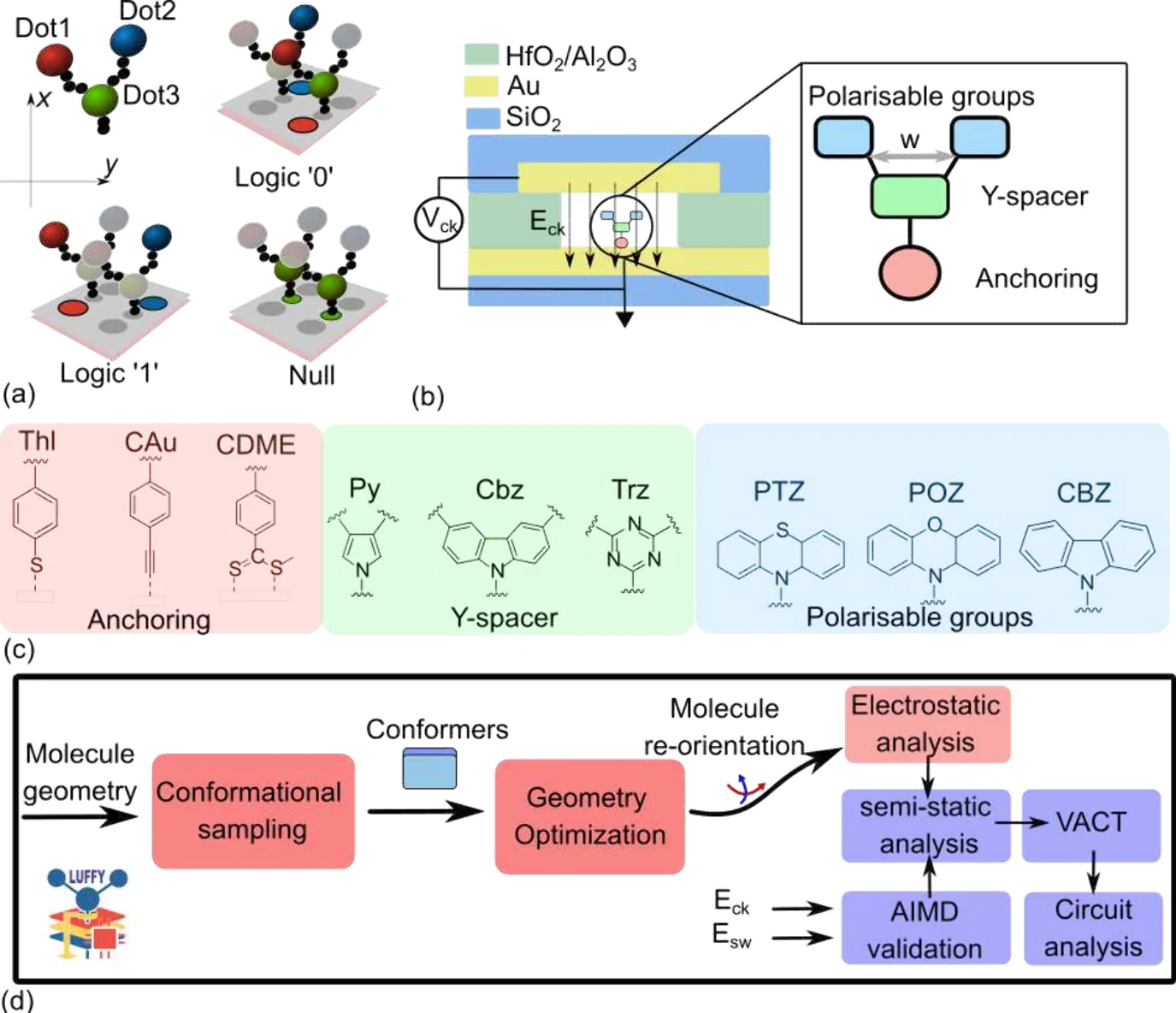
(a) Molecular Field-Coupled Nanocomputing (MolFCN) encodes binary states through charge localization in molecular sites (“dots”), with signal propagation arising from intermolecular electrostatic interactions. Vertical clock fields *E*_*ck*_ enable switching between the *Hold* and *Null* configurations. (b) Dielectric nano-trench architecture proposed for MolFCN implementation: molecules are confined between a ground electrode and a top electrode implementing *E*_*ck*_. (c) Modular AG–SP–PG molecular design employed in this work: anchoring groups (AG) ensure stable and oriented surface binding, spacers (SP) enforce the Y-shaped geometry defining intramolecular dot separation, and polarizable or redox-active groups (PG) enable field-induced charge redistribution. (d) Overview of the unified workflow developed in this Letter, integrating conformational sampling, DFT-based electrostatic analysis, VACT derivation, AIMD-based validation of time-averaged dipole responses under fixed applied fields, and SCERPA-level evaluation to identify and screen MolFCN molecular candidates.

In this architecture, molecules are confined between a ground and a top electrode establishing *E*_*ck*_, essential for polarizing the molecule and enabling information propagation.^6^ MolFCN requires molecular species with robust surface anchoring, controlled orientation, well-separated charge-localization sites, low intrinsic dipole moment, high polarizability, and chemical stability.^8–11^ Identifying and engineering molecular structures that simultaneously satisfy all these requirements remains a major open challenge, and one of the main reasons why a fully functional MolFCN prototype has not yet been realized.^7^ Several possibilities were attempted to address these limitations, including mixed-valence complexes, Y-shaped architectures such as bis-ferrocene, and zwitterions.^12–16^ Their suitability could be evaluated at the circuit level using QCADesigner,^17^ which models information propagation through simplified bistable cells valid for qualitative studies. Instead, physically-precise analyses can be performed using the Self-Consistent ElectRostatic Potential Algorithm (SCERPA), which is embedded within the MoSQuiTo simulation framework.^18,19^ MoSQuiTo explicitly incorporates molecular electrostatics by deriving Voltage-to-Aggregated-Charge Transcharacteristics (VACT) from *ab initio* calculations:^20^ each molecule is represented by three effective point charges—aggregated charges (ACs)—that reproduce its electrostatic response under the applied *E*_*ck*_ and the input potential (*V*_*in*_), defined as the potential difference between the two logical dots.^21^ Within this framework, SCERPA computes circuit-level information propagation by self-consistently determining all intermolecular electrostatic interactions. This method has been validated against full DFT benchmarks, demonstrating *ab initio*-comparable electrostatic accuracy while significantly reducing the computational cost.^22^

Prior analyses largely relied on single, energy-minimized geometries and static assumptions, neglecting conformational diversity, vibrational motion, and field-induced structural responses. However, under realistic conditions, molecules continuously sample a geometric distribution due to thermal fluctuations, synthetic variability, and external fields, significantly affecting charge localization, thus information stability and propagation. These factors must therefore be considered to advance toward an experimental prototype realistically. In molecular electronics, both molecular conformations and electron–vibration coupling critically affect charge localization and functional switching behavior.^23,24^ The absence of methodologies considering these effects represents a key bottleneck in identifying viable MolFCN molecular candidates. In this Letter, we propose the Layered Unified Framework for molFCN systematic analYsis (LUFFY), a VACT-based workflow that enables realistic and device-compatible molecular screening. First, *conformational sampling* identifies low-energy molecular configurations, then refined via *geometry optimization*. These optimized structures undergo *single-point electrostatic analysis* to evaluate their ground-state charge distribution and polarization response. Next, VACTs are extracted using perturbation-grouping-based models. Finally, *ab initio* molecular dynamics (AIMD) assesses molecular stability and responses to different *E*_*ck*_ values. Finally, energy-weighted averaging is applied to obtain conformer/vibration-aware VACTs, and validates switching robustness through dynamic field-driven response simulations. Accordingly, this framework provides a physically grounded and scalable basis for identifying and engineering optimal MolFCN molecular candidates.

We apply LUFFY to a newly constructed dataset of 27 synthetically accessible Y-shaped compounds (M1–M27) designed for MolFCN. Based on their systematic analysis, 5 additional derivatives (M28–M32) were designed to demonstrate LUFFY’s capability for rational and iterative molecular engineering, yielding a final dataset of 32 molecules. Each molecule follows a modular architecture (Figure 1(c)): an anchoring group (AG) enables stable surface binding, a Y-spacer (SP) enforces the required dipole geometry, and a polarizable or redox-active group (PG) provides charge localization and field-driven redistribution (Figure 1(c)). Thiols (Th), ethynyl–Au bonds (CAu), and carbodithioate methyl esters (CDME) were selected as AGs for their strong and stable gold coordination, with differing degrees of lateral mobility.^25–27^ Pyrrole (Py), carbazole (Cbz), and triazole (Trz) serve as SPs, spanning from conjugated and synthetically accessible (Py) to larger footprints (Cbz) and rigid geometries with reduced conformational freedom (Trz).^28–31^ Carbazole (Cbz), phenoxazine (POZ), and phenothiazine (PTZ) act as PGs, offering established redox behavior and tunable polarizability.^29,32,33^ Conformational sampling in implicit water was performed using CREST, retaining structures within 6 kcal/mol of the global minimum.^34–36^ CREST plays a crucial role in LUFFY by efficiently sampling both low- and high-energy conformers with a level of complexity beyond that of well-known conformational search methods based on classical force fields.^37,38^ This advantage arises from its more accurate treatment of long-range interactions (electrostatics and dispersion) and its ability to incorporate solvent effects. CREST integrates the semi-empirical extended tight-binding method GFN2-xTB^34^ with a metadynamics-based search algorithm. Here, we employed the analytical linearized Poisson–Boltzmann (ALPB) implicit solvation model with water parameters,^39^ as water is the most extensively studied solvent in organic chemistry. Nevertheless, other solvents can be considered if additional information about the synthesis or processing conditions is available.^40,41^ The lowest-energy conformers for each species are reported in Figure S1 in the Supplementary Information (SI). The resulting conformers were subsequently optimized in the gas phase at the CAM-B3LYP-D4/def2-TZVP level using ORCA 5.0 ^42–45^ and analyzed in both their neutral and oxidized states. Consequently, the electrostatic characterization reflects the vacuum-like operating conditions expected for MolFCN devices. From their charge distributions, conformer-aware semi-static *V*_*in*_–*μ* transcharacteristics were obtained and mapped to three-point-charge representations. These were then validated through AIMD simulations under fixed applied fields by comparing the resulting time-averaged dipoles with the semi-static predictions, and subsequently incorporated into SCERPA^18–20,22^ to evaluate information propagation under realistic intermolecular coupling. LUFFY thus provides a physically grounded route for systematic MolFCN candidate screening, bridging molecular design, electrostatic modeling, and device-level validation.

Understanding correlations in the high-dimensional property space of the proposed MolFCN candidates can provide valuable insights for the rational design of novel compounds.^46,47^ This strategy has already proven effective for drug-like molecules^48,49^ and e-nose molecular building blocks.^50^ Accordingly, we now explore the pairwise relationship between the total dipole moment |*μ*| and the isotropic polarizability |*α*| in the oxidized form (Figure 2(a)). Each point in the resulting distribution corresponds to a distinct conformer in the dataset. Cbz-based compounds generally display the highest |*μ*| and |*α*| values and the largest conformational variability, whereas Pyr yields lower values and Trz lies in between. The *μ*_*x*_–|*μ*_*y*_| distribution in Figure 2(b) further shows that *μ*_*x*_ is predominantly positive, indicating a natural tendency toward the *Hold* configuration with polarization toward the PGs. At the same time, the lateral component *μ*_*y*_ encodes the *logical state*, as it reflects the charge imbalance between the two PG branches. Since |*μ*_*y*_| is generally non-zero, most species exhibit a *pre-biased logical state* at rest. The impact of this intrinsic asymmetry must be evaluated during VACT characterisation and signal propagation at the circuit level. Pyr-linked compounds display the lowest |*μ*_*y*_|, while Cbz-linked systems show a broader spread; in these compounds, the parallel increase of *μ*_*x*_ and |*μ*_*y*_| suggests that stronger Hold polarization is accompanied by increased asymmetry between the two PG arms, leading to preferential localization on one logical branch. A further analysis of chemical groups has determined that the spacer primarily sets the geometric scale and polarizability, while the anchoring group biases dipole orientation (see Figures S2–S4). The balance between *μ*_*x*_ and *μ*_*y*_ determines whether a molecule is naturally neutral with respect to logic encoding or intrinsically favors one of the two logical states: specifically, *μ*_*x*_ defines the native Hold/Reset behavior, whereas *μ*_*y*_ sets the intrinsic logical state (0 or 1).

**Figure 2:**
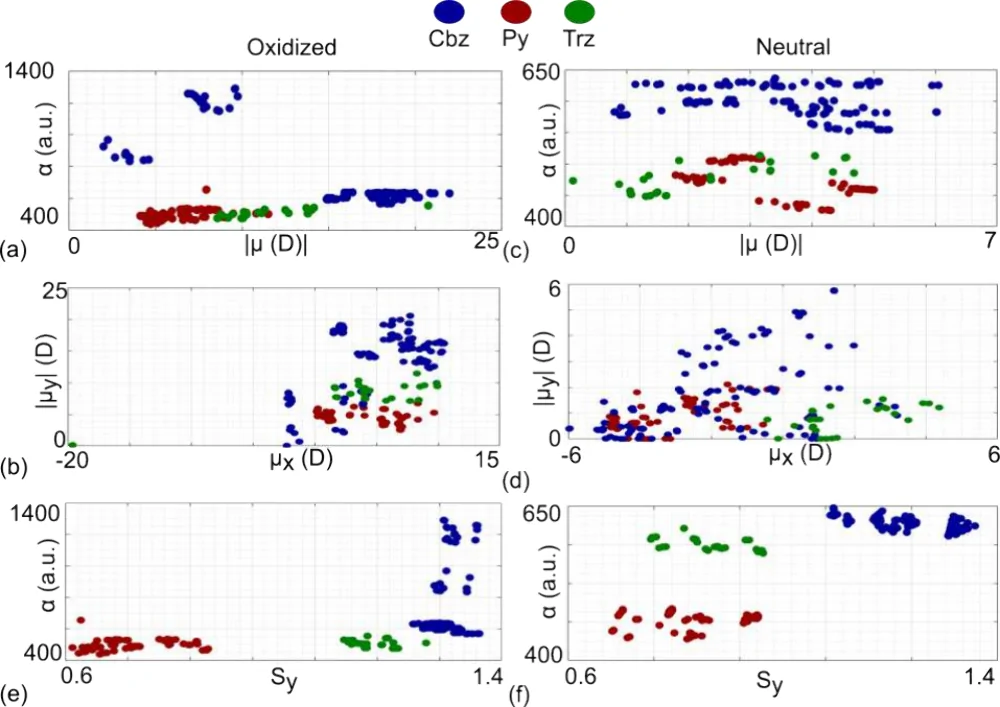
Conformational electrostatic and geometric descriptors for the molecular dataset. (a) Total dipole moment |*μ*| versus isotropic polarizability |*α*| (oxidized state), with each point representing a distinct conformer; Cbz-linked systems show higher |*μ*|, |*α*|, and conformational variability. (b) *μ*_*x*_ versus |*μ*_*y*_| (oxidized state), where predominantly positive *μ*_*x*_ indicates a natural *Hold* configuration; Cbz-linked compounds exhibit increased asymmetry between the two PG branches. (c) |*μ*| versus *α* in the neutral state, with Cbz remaining the most polarizable. (d) *μ*_*x*_ versus |*μ*_*y*_| in the neutral state, showing a generally *Null* configuration (*μ*_*x*_ < 0) and reduced dipole dispersion. (e) Specularity coefficient *S*_*y*_ for the oxidized conformers, where Pyr-linked compounds show higher symmetry and Cbz-linked ones greater geometric asymmetry. (f) *S*_*y*_ versus *α* for neutral conformers, displaying the same spacer-dependent clustering with shifted absolute polarizabilities.

For the neutral compounds, Figure 2(c) shows *α* in the 400–650 a.u. range with no clear correlation to |*μ*|, though Cbz-linked species remain the most polarizable, while Figure 2(d) shows a weaker *μ*_*x*_–|*μ*_*y*_| trend due to the narrower dipole distribution. Most neutrals exhibit negative *μ*_*x*_, indicating a natural *Null* configuration with no charge polarization toward the PGs. Accordingly, subsequent analyses focus on the oxidized species, whose predominantly positive *μ*_*x*_ favors a natural *Hold* configuration and reduces the clock field required relative to the neutral species.^20,51^ Comparison of Figures S2 and S5 shows that oxidation has only a limited effect on the structural descriptors *w* and *α*, while substantially altering the magnitude and orientation of the molecular dipole. The subgroup analyses in Figures S3–S4 and S6–S7 further indicate that the observed trends are primarily governed by molecular geometry and composition rather than by the oxidation state alone. To quantify deviations from mirror symmetry across conformers, we introduce the specularity coefficient *S* (Sec. 6 in SI)­

1 $$S_{x,y,z}=\frac{1}{N}\overset{N}{\underset{i=1}{\sum }}||{\mathrm{r⃗}}_{i}-{\mathrm{r⃗}}_{i}^{\left(x,y,z\right)}||$$

where *N* is the number of atoms in the molecule, **r**_*i*_ is the position vector of atom *i*, and $r_{i}^{\left(x,y,z\right)}$ is the position of the corresponding atom after reflection with respect to the (*x*, *y*, *z*) symmetry plane. Lower values of *S*_*x*,*y*,*z*_ indicate a higher degree of mirror symmetry with respect to the selected plane. As shown in Figures 2(e,f), Pyr-linked compounds generally exhibit lower *S*_*y*_, with the two PG branches remaining nearly specular. Cbz- and, to a lesser extent, Trz-linked compounds display larger *S*_*y*_, reflecting more pronounced Y-shaped and planar arrangements with increased conformational flexibility.

Cbz-linked compounds emerge from this analysis as the most promising candidates, as they combine large PG–PG separation, high polarizability, and broad conformational flexibility. In contrast, Trz-linked systems are more rigid, exhibit fewer accessible conformers, and show lower polarizability (*α*). Pyr-based species display small *w* and *α*, yet remain attractive due to their synthetic accessibility. To improve the performance of Pyr and Trz derivatives, a phenyl unit was introduced between SP and PG (compounds **M28**–**M32**, Figure S8), motivated by the intrinsically high *π*-electron polarizability of phenyl rings. Overall, this analysis demonstrates that phenyl insertion provides a simple and modular strategy to enhance polarizability, tune dipole symmetry, and modulate Y-shaped geometry, thereby yielding more suitable candidates for MolFCN operation (Figures S9–S11).

Building on the electrostatic characterization described above, we perform the *V*_*in*_–*μ* analysis used for VACT extraction. This analysis provides the key figure of merit that quantifies the molecular response to an external electric field and enables its coarse-grained representation as a system of three aggregated charges. The conventional procedure assumes a fixed molecular geometry under the applied *E*_*ck*_ and *V*_*in*_. In practice, this is implemented by embedding the molecule in a four-point-charge environment (Figure 3(a)), thereby neglecting field-induced nuclear rearrangements. To overcome this limitation, we introduce a semi-static approach in which the molecular geometry is re-optimized under the applied *E*_*ck*_ for each value of *V*_*in*_, allowing the structure to adapt to the external fields. Representative results are shown in Figure 3(b–c) for **M6**, identified as one of the most promising MolFCN candidates. For this molecule, the semi-static treatment produces a substantially sharper *V*_*in*_–*μ*_*y*_ transition and a stronger modulation of the dipole response under clocking fields than the static approximation. By allowing nuclear rearrangement under the applied fields, the molecule can relax toward geometries that better stabilize the charge distribution induced by *V*_*in*_, thus providing a more physically accurate description of the transitions between different polarization states. Additional results for **M15**, **M14**, **M9**, and **M29** are provided in Figures S12–S13. Generally, molecules containing highly polarizable spacer units benefit most from the semi-static treatment, whereas less flexible candidates exhibit only minor differences relative to the static approximation. Consequently, the semi-static approach is adopted throughout the remainder of this work.

**Figure 3:**
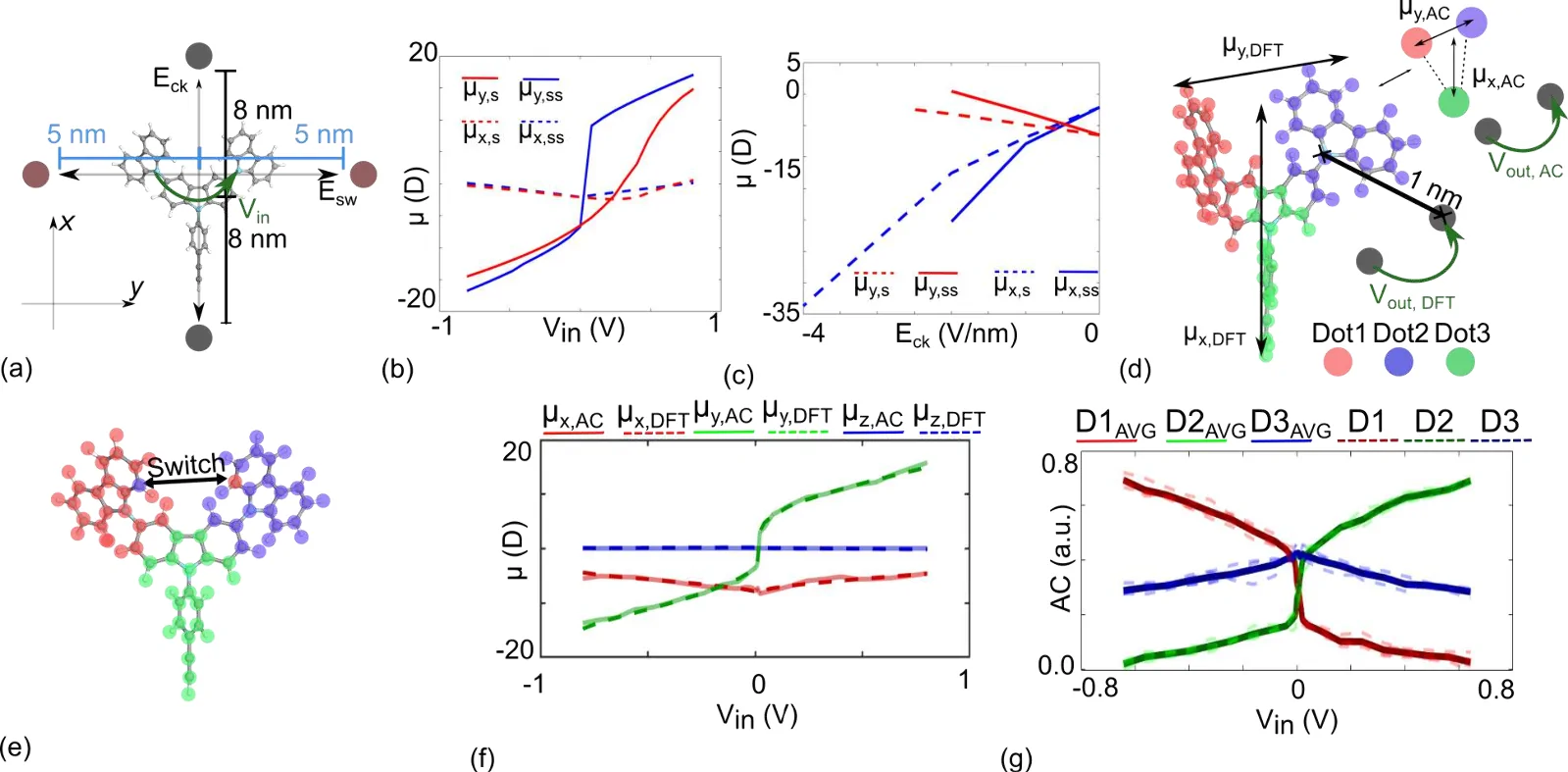
Analysis of **M6** with static, semi-static, and perturbation-based AC models. (a) Conventional static scheme for VACT extraction. (b–c) Comparison of *V*_*in*_–*μ*_*y*_ characteristics: the static case (*μ*_*s*_) shows a smooth response, while the semi-static case (*μ*_*ss*_) yields a sharp transition near *V*_*in*_ = 0, strongly impacting MolFCN applicability. (d) *E*_*ck*_–*μ*_*x*,*y*_ curves at *V*_*in*_ = 0, highlighting deviations of ∼10 D at −2 V/nm. (e) Schematic of the perturbation-based optimization, used to overcome the rigidity of conventional spatial grouping. (f) Validation of the perturbation-based AC model for the lowest-energy conformer, reproducing *V*_*in*_–*μ* characteristics obtained through DFT (dashed lines). (g) Boltzmann-weighted average of conformer dipole characteristics reconstructed using the perturbation-based charge-grouping method. The semi-static approach provides a more realistic description of field-induced molecular relaxation, while the perturbation-based AC and averaged VACT schemes ensure accurate reproduction of dipole components, *V*_out_, and conformational diversity (see SI for details).

To enable efficient circuit-level simulations within MoSQuiTo,^21^ the molecular electrostatic response must be translated into VACTs. The conventional extraction procedure relies on a fixed spatial partitioning of the molecular charge distribution into three aggregated charges (Figure 3(d)). However, because this grouping cannot adapt to field-induced charge redistribution, it may introduce significant inaccuracies. To mitigate this limitation, we developed a perturbation-based grouping optimization in which the conventional spatial grouping is used as an initial guess and atomic charges are iteratively exchanged among the aggregated charges. Candidate groupings are accepted only if they reduce the deviation from the DFT electrostatic response, yielding an optimized three-point-charge representation (Figure 3(e), further details in SI). The methodology was validated across representative molecular candidates and operating conditions. Table 1 compares the mean absolute dipole errors obtained with the conventional spatial grouping and the perturbation-based method. The error is evaluated between VACT- and DFT-derived dipoles, averaging first over the sampled *V*_*in*_ values and then over all conformers. In all cases, the optimized grouping markedly reduces the reconstruction error, generally reaching sub-Debye accuracy. For **M6**, Figure 3(f) shows that the resulting three-point-charge model closely reproduces the DFT *V*_*in*_–*μ* characteristics. Further details on the error calculation are provided in the SI.

**Table 1:** Semi-Static Dipole-Component Errors (*e*_*x*_, *e*_*y*_, *e*_*z*_) in Debye for Selected Compounds in the Oxidized State^a^

Mol.	*E*_*ck*_	*e*_*x*,1*st*,*p*_ (D)	*e*_*y*,1*st*,*p*_ (D)	*e*_*x*,p_	*e*_*y*,p_	*e*_*z*,p_	*e*_*x*,sp_	*e*_*y*,sp_	*e*_*z*,sp_
4	0.00	0.13	1.20	0.22	0.84	0.93	6.22	2.95	1.24
29	0.00	0.19	0.95	0.15	0.81	0.20	4.26	2.72	0.41
28	0.00	0.16	0.44	0.22	0.73	1.30	7.30	3.21	1.73
13	0.00	0.14	1.38	0.16	0.86	0.84	4.59	2.80	0.89
15	0.00	0.23	0.36	0.23	0.43	0.17	4.82	3.84	0.20
15	–1.00	0.14	0.52	0.18	0.68	0.36	0.58	3.59	0.36
									
6	0.00	0.24	0.60	0.24	0.57	0.76	6.35	3.49	0.44
6	–1.00	0.32	0.35	0.28	0.48	0.66	1.63	2.77	0.58
6	–1.50	0.23	0.54	0.30	0.48	0.63	1.83	2.15	0.71
6	–2.00	0.59	0.55	0.73	0.51	0.57	7.20	1.36	0.87

a Columns report, side-by-side, the first-conformer errors (*e*_*x*,1*st*,*p*_, *e*_*y*,1*st*,*p*_) using perturative method, the average perturbation-based grouping error (*e*_*x*,*p*_, *e*_*y*,*p*_, *e*_*z*,*p*_), and the spatial grouping (*e*_*x*,*sp*_, *e*_*y*,*sp*_, *e*_*z*,*sp*_).

Because different conformers may exhibit distinct intrinsic dipoles and switching characteristics, conformer-dependent VACTs were also investigated. Ensemble VACTs representative of thermally accessible conformations were constructed through Boltzmann-weighted averaging,

2 $$\left\langle AC_{dot,j}\right\rangle =\overset{N_{conf}}{\underset{i=1}{\sum }}w_{i}AC_{dot,j,i},,w_{i}=\frac{\exp\left(-\Delta E_{i}/k_{B}T\right)}{\overset{N_{conf}}{\underset{l=1}{\sum }}\exp\left(-\Delta E_{l}/k_{B}T\right)}$$

where *AC*_dot,*j*,*i*_ is the aggregated charge associated with dot *j* of conformer *i*, *w*_*i*_ is its Boltzmann weight, *N*_conf_ is the number of conformers, Δ*E*_*i*_ = *E*_*i*_ – *E*_min_ is the energy relative to the lowest-energy conformer, *k*_B_ is the Boltzmann constant, and *T* is the temperature. Across multiple cases (Figure 3(g) and Figures S14–S16), the resulting averaged VACTs faithfully reproduce the DFT response while naturally accounting for conformational diversity, including conformers with different native charge states. This finding raises an important modeling question: *should the representative VACT be derived from the lowest-energy conformer or from a thermally weighted conformational ensemble?*. Figures S14 and S15 show that the effect of conformational averaging is strongly molecule dependent. For some candidates, the Boltzmann-weighted average closely follows the individual conformer characteristics, whereas for others it yields intermediate *V*_in_–*μ* transcharacteristics lying between conformers with different native dipole polarizations. Determining which representation is more physically meaningful therefore requires an independent comparison with the molecular response under dynamical operating conditions.

Figure 4 compares the *V*_in_–*μ* characteristics of representative molecular candidates: **M6** in panels (a)–(d), **M15** in (e), and **M28** in (f). AIMD simulations were initialized from the lowest-energy CREST conformer using a 0.3 fs time step. Initial velocities were generated at 300 K, and the temperature was maintained at 300 K using a Berendsen thermostat with a 10 fs time constant. The resulting time-averaged dipoles were compared with the ensemble-averaged and lowest-energy-conformer *V*_in_–*μ* characteristics. AIMD simulations were performed at fixed *V*_in_ = −0.32, 0, and +0.32 V, probing the molecular response around the intermediate operating point and under both saturation conditions. The corresponding deviations, *ε*_avg_ and *ε*_1st_, are reported in Table 2.

**Figure 4:**
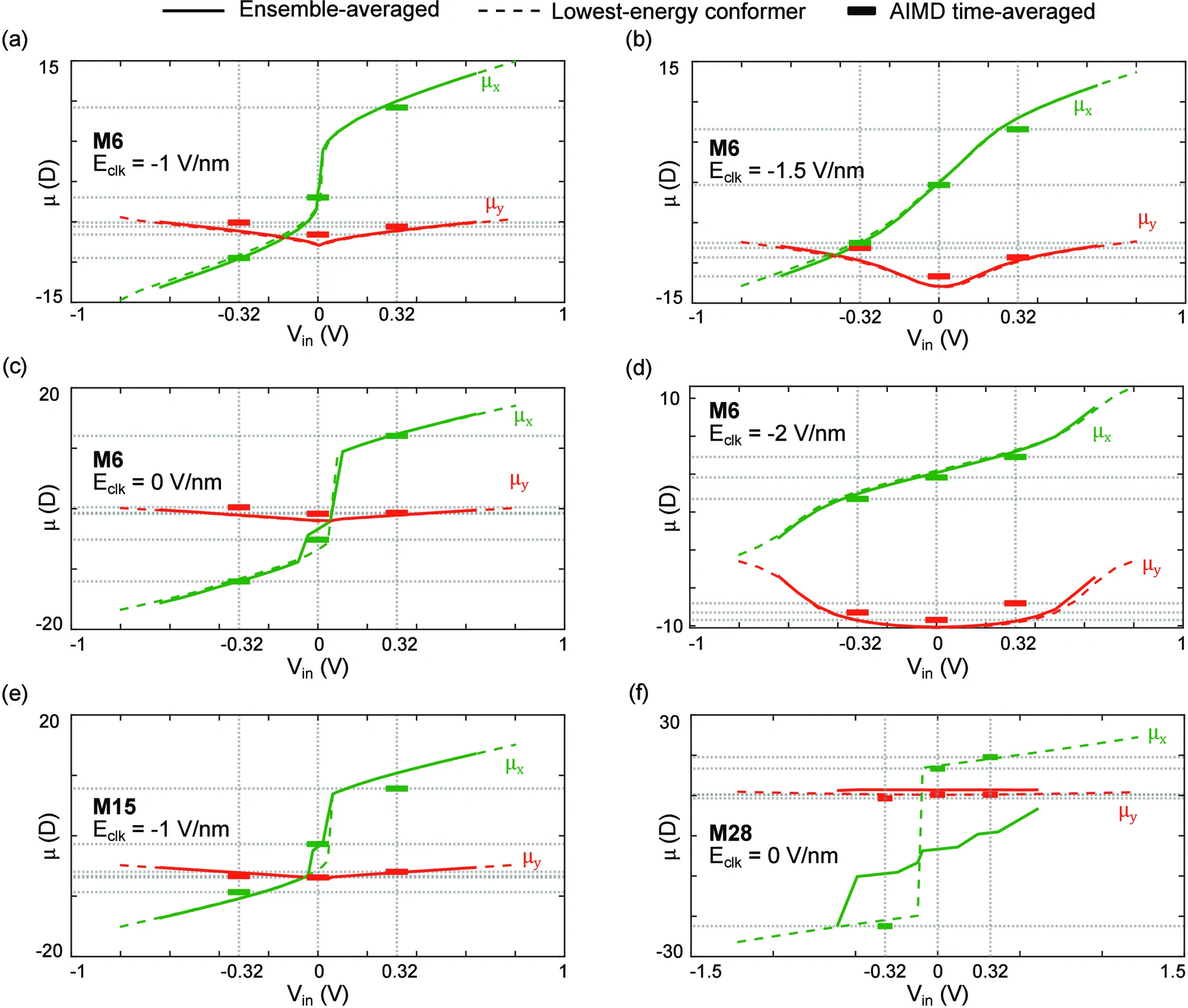
Comparison between the semi-static *V*_in_–*μ* characteristics and AIMD time-averaged dipoles for representative molecular candidates **M6**, **M15**, and **M28**. Solid and dashed curves represent the ensemble-averaged and lowest-energy-conformer *V*_in_–*μ* characteristics, respectively. Green and red denote the *μ*_*x*_ and *μ*_*y*_ components of the dipole moment, respectively. Rectangular markers indicate the corresponding AIMD time-averaged dipoles at *V*_in_ = −0.32, 0, and +0.32 V. (a)–(d) **M6** under clock fields of *E*_*ck*_ = −1, −1.5, 0, and −2 V/nm, respectively. (e) **M15** at *E*_*ck*_ = −1 V/nm. (f) **M28** at *E*_*ck*_ = 0 V/nm.

**Table 2:** Absolute Errors *ϵ* = |*μ* ^AIMD^ – *μ* ^ref^| (Debye) between Time-Averaged AIMD Dipoles (*μ* ^AIMD^) and Reference Values from the *V*_*in*_–*μ* Curves^a^

			*ϵ*_*avg*_ (D)			*ϵ*_1*st*_ (D)		
Mol.	*E*_*ck*_ (V/nm)	*V*_*in*_ (V)	*x*	*y*	*z*	*x*	*y*	*z*
6	–1	–0.32	1.16	0.11	1.25	1.20	0.33	0.19
		0.00	1.28	0.74	1.01	1.34	0.92	0.06
		+0.32	0.51	0.80	0.61	0.59	0.81	0.29
	–1.5	–0.32	1.54	0.13	0.81	1.70	0.26	0.25
		0.00	1.24	0.29	0.76	1.32	0.40	0.38
		+0.32	0.38	1.43	0.92	0.51	1.43	0.03
	0	–0.32	1.29	0.02	1.41	1.39	0.42	0.29
		0.00	1.11	1.73	1.09	1.24	1.63	0.02
		+0.32	0.40	0.30	0.63	0.47	0.25	0.28
	–2	–0.32	1.04	0.66	1.05	1.11	0.95	0.28
		0.00	0.91	0.64	0.98	1.02	0.89	0.42
		+0.32	2.35	0.72	1.17	2.56	0.90	0.21
								
15	–1	–0.32	0.57	1.09	0.17	0.52	1.01	0.58
		0.00	0.15	0.49	0.07	0.17	4.28	0.41
		+0.32	0.12	2.57	0.43	0.12	2.55	0.04
								
28	0	–0.32	2.12	13.05	1.43	0.98	1.49	1.12
		0.00	1.18	20.05	0.06	0.04	0.66	0.36
		+0.32	1.17	18.80	0.44	0.01	0.43	0.56

a Two sets of reference values are considered: the energy-averaged curve, leading to *ϵ*_*avg*_, and the first conformer, thus computing *ϵ*_1*st*_.

For **M6** at *E*_*ck*_ = −1 and −1.5 V/nm (Figure 4(a,b)), both the ensemble-averaged and lowest-energy-conformer *V*_*in*_–*μ* curves reproduce the AIMD steady-state dipoles with comparable accuracy. At *V*_*in*_ = +0.32 V, the dipole reaches equilibrium only after ∼100 fs, whereas faster stabilization is observed at *V*_*in*_ = 0 and −0.32 V (Figure S17­(a)–(f)). This behavior originates from the initial opposition between the native *μ*_*y*_ and the field-induced dipole, requiring structural reorganization before convergence. Such transient dynamics directly determine the intrinsic switching-speed limits of MolFCN devices.^52,53^ On the other hand, for **M6** at *E*_*ck*_ = 0 V/nm (Figure 4(c)), a larger deviation is observed for *μ*_*y*_ at *V*_*in*_ = 0 V, where *ε*_*y*,avg_ and *ε*_*y*,1st_ both exceed 1 D. At *V*_*in*_ = ±0.32 V, the errors become comparable as the system approaches saturation and the semi-static *V*_*in*_–*μ* characteristics converge toward the AIMD averages (Figure S17­(g)–(i)). Moreover, for **M6** at *E*_*ck*_ = −2 V/nm (Figure 4(d)), *ϵ*_avg_ and *ϵ*_1st_ at *V*_*in*_ = 0 V remain nearly identical, while the residual deviation in *μ*_*x*_ originates from the ∼150 fs relaxation needed to reach ≈−20 D (Figure S17­(l)); at such values of electric field, *μ*_*y*_ is largely quenched while *μ*_*x*_ stabilizes a robust *Null* state. A similar settling behavior occurs at *V*_*in*_ = ±0.32 V (Figure S17­(m),(n)), where the dipoles converge close to the semi-static prediction, with residual errors of *ϵ*_*x*,avg_ = 1.04, *ϵ*_*y*,avg_ = 0.66 at *V*_*in*_ = −0.32 V, and *ϵ*_*x*,avg_ = 2.35, *ϵ*_*y*,avg_ = 0.72 at *V*_*in*_ = +0.32 V, reinforcing that finite settling times must be accounted for when estimating clock frequency. Comparable behavior is observed for **M15** at *E*_*ck*_ = −1 V/nm (Figure 4(e), Figure S18­(a)–(c)): at *V*_*in*_ = 0 V, AIMD reveals large *μ*_*y*_ oscillations while *μ*_*x*_ remains stable, and at *V*_*in*_ = ±0.32 V both dipole components agree well with the ensemble-averaged semi-static model. Notably, here the ensemble-averaged *V*_*in*_–*μ*_*y*_ reproduces the AIMD time average more accurately than the first-conformer curve, indicating that incorporating conformational sampling leads to a more accurate semi-static model.

The opposite trend is observed for **M28** (Figure 4(f)): at *E*_*ck*_ = 0 V/nm, the ensemble-averaged *μ*_*x*,*y*_ curves (black) deviate from the AIMD time-averaged dipoles at *V*_*in*_ = 0, −0.32, and +0.32 V (see the corresponding *μ*(*t*) traces in Figure S19­(a)–(c)). In contrast, the *V*_*in*_–*μ* characteristics of the lowest-energy conformer (light brown in Figure 4(f)) closely reproduce the AIMD averages, as reflected by the lower *ϵ*_1st_ values reported in Table 2. This behavior is consistent with the increased structural rigidity associated with the phenyl linker, which limits conformational fluctuations during AIMD and makes the lowest-energy conformer more representative of the time-averaged molecular response. Taken together, these results show that agreement between AIMD time-averaged dipoles and the semi-static model provides a physically grounded criterion for selecting the most representative VACT. Accordingly, the ensemble-averaged or lowest-energy-conformer VACT with the smallest deviation from the AIMD response is used in subsequent MolFCN simulations. The transient dipole dynamics thus validate the semi-static information-propagation framework over the relevant stabilization time scales.

Finally, we use the AIMD-validated semi-static averaged VACTs for **M6** at *E*_*ck*_ = −1, −1.5, and −2 V/nm as inputs to SCERPA, as these clock-field conditions provide stable polarization states suitable for robust information propagation. The colored dots represent the electrostatic potential evaluated 0.2 nm above the upper molecular sites, with brighter colors indicating localization of the oxidized charge. The wire comprises three clocking zones of eight molecules each, spaced by 1 nm and driven by the signals in Figure S20­(a). Figure 5(a) shows three propagation steps for logical inputs ‘0’ and ‘1’, demonstrating correct transfer of the logic state from the first to the final cell. The alternating upper and lower polarization follows the QCA information-propagation mechanism illustrated in Figure 1(a), with the output read from the polarization state of the final cell. Full propagation traces are provided in Figure S20. This result completes the first systematic validation workflow of a MolFCN molecular candidate, spanning conformational and electrostatic characterization, semi-static modeling, dynamical validation, and circuit-level propagation, and demonstrates the strong potential of **M6** for MolFCN architectures. Figure 5(b) shows a minimal six-cell wire based on **M28**. Owing to the intrinsic VACT asymmetry (Figure 4(f)), which favors one logic state, the intermolecular spacing was reduced to 0.8 nm to enhance electrostatic coupling. Using the lowest-energy-conformer VACT validated at *E*_*ck*_ = 0 V/nm, where **M28** naturally exhibits a stable *Hold* configuration, and applying ± 1.3 V to the drivers, we observe the correct propagation of logical ‘0’ and ‘1’. This result constitutes a first, preliminary demonstration of information transport using **M28**, showing that, despite its native VACT asymmetry, it remains a promising candidate for MolFCN implementations.

**Figure 5:**
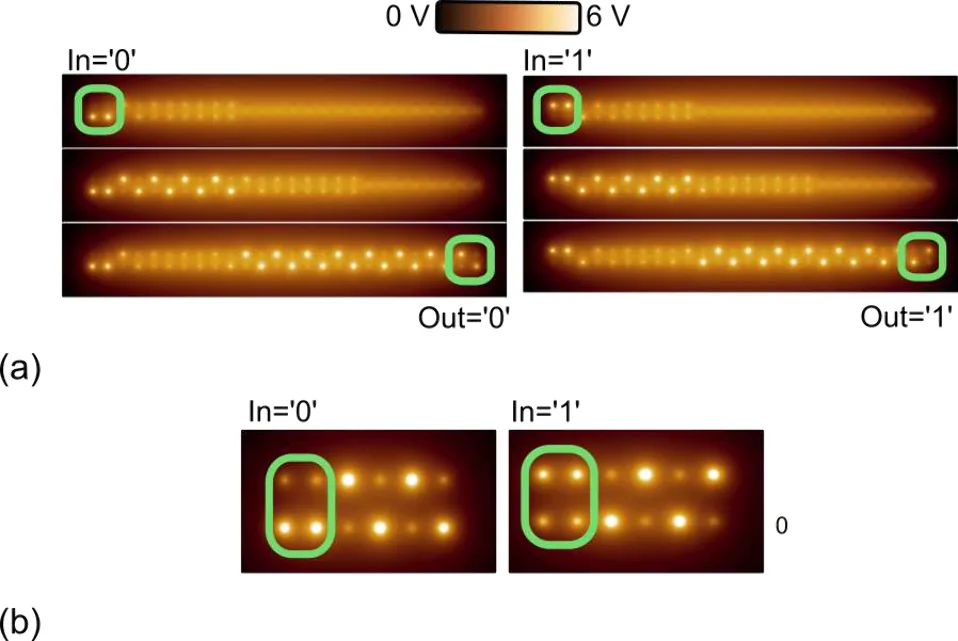
SCERPA information propagation for the selected MolFCN candidates. The colored dots represent the electrostatic potential evaluated 0.2 nm above the plane of the upper molecular sites, with brighter regions indicating positive potential and preferential localization of the oxidized charge. The alternating upper and lower polarization follows the QCA information-propagation mechanism illustrated in Figure 1(a), while the logical output is read from the polarization state of the final molecular cell. (a) Propagation of logical inputs ‘0’ and ‘1’ through three eight-molecule clocking zones based on **M6**, with 1 nm intermolecular spacing and driven by the signals reported in Figure S20­(a). (b) Minimal six-cell wire based on **M28**, with the spacing reduced to 0.8 nm to compensate for its intrinsic VACT asymmetry, showing propagation under ± 1.3 V drivers. The electrostatic-potential scale is reported by the color bar.

Overall, our work provides the first framework for the rational design and validation of molecular candidates for MolFCN architectures, bridging quantum-informed molecular properties with functional device behavior. By explicitly accounting for conformational variability and its effect on electrostatic response, LUFFY enables the consistent and accurate identification of molecules capable of supporting stable information transport. Applied to a dataset of 27 compounds and 5 rationally engineered derivatives, the framework identifies carbazole-based Y-spacers as the most promising class and, crucially, reveals two molecular candidates whose electrostatic transcharacteristics remain robust across conformations. When introduced into SCERPA, these candidates enable reliable information propagation. These findings demonstrate that conformer-aware electrostatic descriptors, combined with AIMD-based selection of the most representative molecular response, are essential for predicting functionality in MolFCN systems. In addition, the systematic advantage of the oxidized state further refines the molecular design space.

Beyond candidate identification, LUFFY establishes a transferable strategy for connecting molecular design with circuit-level functionality and can be extended to any three-dot molecular architecture, including the widely investigated transition-metal-based candidates. Future developments will incorporate solvent- and substrate-induced effects, including substrate-aware VACTs, to enable more realistic modeling of molecule–environment interactions. The framework is also inherently compatible with sustainable machine learning practices^54^ and can be extended to automate the extraction of optimal transcharacteristics and accelerate high-throughput screening, thereby substantially reducing the experimental search space toward a functional prototype. Together, these advances pave the way toward predictive, scalable, and AI-assisted discovery of MolFCN building blocks.

## Supplementary Material



## Data Availability

The dataset generated in this work and scripts to use the LUFFY workflow are available in the LUFFY GitHub repository (https://github.com/federicoravera-sudo/LUFFY).

## Notes

Additional analyses of the dataset introduced in this work are provided in the online Supplementary Information. We also present additional calculations using the LUFFY framework, along with computational details that further support the main conclusions of this work (PDF)

## #

Center for Advanced Systems Understanding (CASUS), Conrad-Schiedt-Straße 20, Görlitz 02826, Germany

## References

[1] Anderson, N. ; Bhanja, S. Field-Coupled Nanocomputing: Paradigms, Progress, and Perspectives; Lecture Notes in Computer Science; Springer Berlin Heidelberg: 2014.

[2] Listo R., Mo F., Ravera F., Ardesi Y., Vacca M., Piccinini G., Krzywiecki M., Vezzoli A., Graziano M. (2025). Unfolding potential
and challenges in molecular field-coupled nanocomputing. Nano Futures.

[3] Lent C. S., Isaksen B., Lieberman M. (2003). Molecular quantum-dot cellular automata. J. Am. Chem. Soc..

[4] Lent C. S., Tougaw P. D., Porod W., Bernstein G. H. (1993). Quantum
cellular automata. Nanotechnology.

[5] Lu Y., Liu M., Lent C. (2007). Molecular
quantum-dot cellular automata: From molecular
structure to circuit dynamics. J. Appl. Phys..

[6] Pulimeno A., Graziano M., Antidormi A., Wang R., Zahir A., Piccinini G. (2014). Understanding
a bisferrocene molecular QCA wire. Field-Coupled
Nanocomputing: Paradigms, Progress, and Perspectives.

[7] Ravera, F. ; Beretta, G. ; Ardesi, Y. ; Krzywiecki, M. ; Graziano, M. ; Piccinini, G. A Roadmap for Molecular Field-Coupled Nanocomputing Actualization. 2023 IEEE Nanotechnology Materials and Devices Conference (NMDC). 2023; pp 212–213.

[8] Ferrero, E. ; Ravera, F. ; Listo, R. ; Ardesi, Y. ; Piccinini, G. ; Graziano, M. Technology and Power-aware Investigation of Nanostructures for Molecular Field-Coupled Nanocomputing. 2025 IEEE Computer Society Annual Symposium on VLSI (ISVLSI). 2025; pp 1–6.

[9] Qi H., Sharma S., Li Z., Snider G. L., Orlov A. O., Lent C. S., Fehlner T. P. (2003). Molecular quantum cellular automata
cells. Electric field driven switching of a silicon surface bound
array of vertically oriented two-dot molecular quantum cellular automata. J. Am. Chem. Soc..

[10] Santana-Bonilla A., Medrano Sandonas L., Gutierrez R., Cuniberti G. (2019). Exploring
the write-in process in molecular quantum cellular automata: a combined
modeling and first-principle approach. J. Phys.:
Condens. Matter.

[11] Blair E. (2019). Electric-field
inputs for molecular quantum-dot cellular automata circuits. IEEE Trans. Nanotechnol..

[12] Lu Y., Lent C. S. (2005). Theoretical study
of molecular quantum-dot cellular
automata. J. Comput. Electron..

[13] Macrae R. M. (2023). Mixed-valence
realizations of quantum dot cellular automata. J. Phys. Chem. Solids.

[14] Liza N., Lu Y., Blair E. P. (2022). Designing boron-cluster-centered zwitterionic Y-shaped
clocked QCA molecules. Nanotechnology.

[15] Groizard T., Kahlal S., Halet J.-F. (2020). Zwitterionic
mixed-valence species
for the design of neutral clocked molecular quantum-dot cellular automata. Inorg. Chem..

[16] Arima V., Iurlo M., Zoli L., Kumar S., Piacenza M., Della Sala F., Matino F., Maruccio G., Rinaldi R., Paolucci F., Marcaccio M., Cozzi P. G., Bramanti A. P. (2012). Toward
quantum-dot cellular automata units: thiolated-carbazole linked bisferrocenes. Nanoscale.

[17] Walus K., Dysart T.J., Jullien G.A., Budiman R.A. (2004). QCADesigner: A Rapid
Design and Simulation Tool for Quantum-Dot Cellular Automata. IEEE Trans. Nanotechnol..

[18] Ardesi Y., Wang R., Turvani G., Piccinini G., Graziano M. (2020). SCERPA: A self-consistent algorithm for the evaluation
of the information propagation in molecular field-coupled nanocomputing. IEEE Trans. Comput.-Aided Des. Integr. Circuits Syst..

[19] Ardesi Y., Turvani G., Graziano M., Piccinini G. (2021). Scerpa simulation
of clocked molecular field-coupling nanocomputing. IEEE Trans. Very Large Scale Integr. (VLSI) Syst..

[20] Ardesi Y., Beretta G., Vacca M., Piccinini G., Graziano M. (2022). Impact of molecular electrostatics
on field-coupled
nanocomputing and quantum-dot cellular automata circuits. Electronics.

[21] Ardesi Y., Pulimeno A., Graziano M., Riente F., Piccinini G. (2018). Effectiveness
of molecules for quantum cellular automata as computing devices. J. Low Power Electron. Appl..

[22] Ardesi Y., Mo F., Vacca M., Piccinini G., Graziano M. (2025). Guesstimation of Molecular
Ensemble Electrostatics Properties Through SCERPA-DFT Calculation:
Molecular Field-Coupled Nanocomputing as a Case Study. Adv. Theory Simul..

[23] Venkataraman L., Klare J. E., Nuckolls C., Hybertsen M. S., Steigerwald M. L. (2006). Dependence of single-molecule junction conductance
on molecular conformation. Nature.

[24] Galperin M., Nitzan A., Ratner M. A. (2007). Molecular
transport junctions: vibrational
effects. J. Phys.: Condens. Matter.

[25] Love J. C., Estroff L. A., Kriebel J. K., Nuzzo R. G., Whitesides G. M. (2005). Self-assembled
monolayers of thiolates on metals as a form of nanotechnology. Chem. Rev..

[26] Herrer L., González-Orive A., Marqués-González S., Martín S., Nichols R. J., Serrano J. L., Low P. J., Cea P. (2019). Electrically transmissive alkyne-anchored monolayers on gold. Nanoscale.

[27] Ward J. S., Vezzoli A., Wells C., Bailey S., Jarvis S. P., Lambert C. J., Robertson C., Nichols R. J., Higgins S. J. (2024). A Systematic
Study of Methyl Carbodithioate Esters as Effective Gold Contact Groups
for Single-Molecule Electronics. Angew. Chem..

[28] Shi T., Yin G., Wang X., Xiong Y., Peng Y., Li S., Zeng Y., Wang Z. (2023). Recent advances in the syntheses
of pyrroles. Green Synth. Catal..

[29] Munir R., Zahoor A. F., Anjum M. N., Nazeer U., Haq A. U., Mansha A., Chaudhry A. R., Irfan A. (2025). Synthesis And Photovoltaic
Performance of Carbazole (Donor) Based Photosensitizers in Dye-Sensitized
Solar Cells (DSSC): A Review. Top. Curr. Chem..

[30] Yamada R., Albrecht K., Ohto T., Minode K., Yamamoto K., Tada H. (2018). Single-molecule rectifiers
based on voltage-dependent deformation
of molecular orbitals in carbazole oligomers. Nanoscale.

[31] Mishra P., Singh N., Satya, Siddique A., Joshi S. (2025). others Multifunctional
Potential of Triazole-based Metal-Organic Frameworks: An Overview. Chem. Inorg. Mater..

[32] Buene A. F., Almenningen D. M. (2021). Phenothiazine and phenoxazine sensitizers for dye-sensitized
solar cells–an investigative review of two complete dye classes. J. Mater. Chem. C.

[33] Luo J.-S., Wan Z.-Q., Jia C.-Y. (2016). Recent advances in phenothiazine-based
dyes for dye-sensitized solar cells. Chin. Chem.
Lett..

[34] Bannwarth C., Ehlert S., Grimme S. (2019). GFN2-xTB—An accurate and broadly
parametrized self-consistent tight-binding quantum chemical method
with multipole electrostatics and density-dependent dispersion contributions. J. Chem. Theory Comput..

[35] Pracht P., Bohle F., Grimme S. (2020). Automated exploration
of the low-energy
chemical space with fast quantum chemical methods. Phys. Chem. Chem. Phys..

[36] Pracht P., Grimme S., Bannwarth C., Bohle F., Ehlert S., Feldmann G., Gorges J., Müller M., Neudecker T., Plett C. (2024). others CREST—A program for
the exploration of low-energy molecular chemical space. J. Chem. Phys..

[37] Medrano
Sandonas L., Van Rompaey D., Fallani A., Hilfiker M., Hahn D., Perez-Benito L., Verhoeven J., Tresadern G., Kurt Wegner J., Ceulemans H., Tkatchenko A. (2024). Dataset for quantum-mechanical exploration of conformers
and solvent effects in large drug-like molecules. Sci. Data.

[38] Hinostroza
Caldas A., Kokorin A., Tkatchenko A., Medrano Sandonas L. (2026). Assessing the performance of quantum-mechanical descriptors
in physicochemical and biological property prediction. Digit. Discov..

[39] Ehlert S., Stahn M., Spicher S., Grimme S. (2021). Robust and Efficient
Implicit Solvation Model for Fast Semiempirical Methods. J. Chem. Theory Comput..

[40] Arima V., Iurlo M., Zoli L., Kumar S., Piacenza M., Della Sala F., Matino F., Maruccio G., Rinaldi R., Paolucci F., Marcaccio M., Cozzi P. G., Bramanti A. P. (2012). Toward
quantum-dot cellular automata units: thiolated-carbazole linked bisferrocenes. Nanoscale.

[41] Christie J. A., Forrest R. P., Corcelli S. A., Wasio N. A., Quardokus R. C., Brown R., Kandel S. A., Lu Y., Lent C. S., Henderson K. W. (2015). Synthesis of a Neutral Mixed-Valence Diferrocenyl Carborane
for Molecular Quantum-Dot Cellular Automata Applications. Angew. Chem., Int. Ed..

[42] Neese F. (2022). Software update:
The ORCA program system—Version 5.0. WIREs Comput. Mol. Sci..

[43] Yanai T., Tew D. P., Handy N. C. (2004). A new hybrid exchange–correlation
functional using the Coulomb-attenuating method (CAM-B3LYP). Chem. Phys. Lett..

[44] Weigend F., Ahlrichs R. (2005). Balanced basis sets of split valence,
triple zeta valence
and quadruple zeta valence quality for H to Rn: Design and assessment
of accuracy. Phys. Chem. Chem. Phys..

[45] Caldeweyher E., Ehlert S., Hansen A., Neugebauer H., Spicher S., Bannwarth C., Grimme S. (2019). A generally applicable
atomic-charge dependent London dispersion correction. J. Chem. Phys..

[46] Butler K.
T., Davies D. W., Cartwright H. M., Isayev O., Walsh A. (2018). Machine learning
for molecular and materials science. Nature.

[47] Fromer J. C., Coley C. W. (2024). An algorithmic framework
for synthetic cost-aware decision
making in molecular design. Nat. Comput. Sci..

[48] Medrano
Sandonas L., Hoja J., Ernst B. G., Vázquez-Mayagoitia Á., DiStasio R. A., Tkatchenko A. (2023). “Freedom of design”
in chemical compound space: towards rational in silico design of molecules
with targeted quantum-mechanical properties. Chem. Sci..

[49] Hilfiker, M. ; Medrano Sandonas, L. ; Klähn, M. ; Engkvist, O. ; Tkatchenko, A. Leveraging Quantum Mechanical Properties to Predict Solvent Effects on Large Drug-Like Molecules. AI in Drug Discovery; Springer: 2025; pp 47–57.

[50] Chen L., Medrano Sandonas L., Huang S., Croy A., Cuniberti G. (2026). Towards the
design of artificial sensing materials via quantum-informed explainable
AI. J. Cheminform..

[51] Ravera F., Ardesi Y., Piccinini G., Graziano M. (2024). Technology-Aware Simulation
for Prototyping Molecular Field-Coupled Nanocomputing. IEEE Trans. Nanotechnol..

[52] Listo, R. ; Ravera, F. ; Beretta, G. ; Ardesi, Y. ; Piccinini, G. ; Graziano, M. Unveiling charge dynamics in molecular field-coupled nanocomputing. 2024 IEEE 24th International Conference on Nanotechnology (NANO). 2024; pp 424–429.

[53] Ardesi Y., Gaeta A., Beretta G., Piccinini G., Graziano M. (2021). Ab initio molecular dynamics simulations
of field-coupled
nanocomputing molecules. J. Integr. Circuits
Syst..

[54] Medrano Sandonas, L. , Perspective: Towards sustainable exploration of chemical spaces with machine learning. arXiv, 2026; https://arxiv.org/abs/2604.00069.

