# Supplementary material for: Toward the Rational Design of Molecular Field-Coupled Nanocomputing Candidates
Source: Nano Lett. 2026 Sep 12;26(37):12359–68. doi: 10.1021/acs.nanolett.6c02459 (PMC13614311; doi:10.1021/acs.nanolett.6c02459)
Supplement: Supplementary file 1 [file nl6c02459_si_001.pdf]

# Supporting Information

## *Toward the Rational Design of Molecular Field-Coupled Nanocomputing Candidates*

Federico Ravera[1,*], Leonardo Medrano Sandonas[2,*], Andrea Vezzoli[3], Yuri Ardesi[1], Mariagrazia Graziano[4], Gianluca Piccinini[1], and Gianaurelio Cuniberti[2,5,6,*]

[1]Department of Electronics and Telecommunication, Politecnico di Torino, Torino 10129, Italy
[2]Institute for Materials Science and Max Bergmann Center of Biomaterials, TUD Dresden University of Technology, Dresden 01062, Germany
[3]Department of Chemistry, University of Liverpool, Liverpool L69 7ZD, United Kingdom
[4]Department of Applied Science and Technology, Politecnico di Torino, Torino 10129, Italy
[5]Cluster of Excellence CARE, TUD Dresden University of Technology and RWTH Aachen, Dresden 01062, Germany
[6]Cluster of Excellence CeTI, TUD Dresden University of Technology, Dresden 01062, Germany

## 1. Molecular Dataset

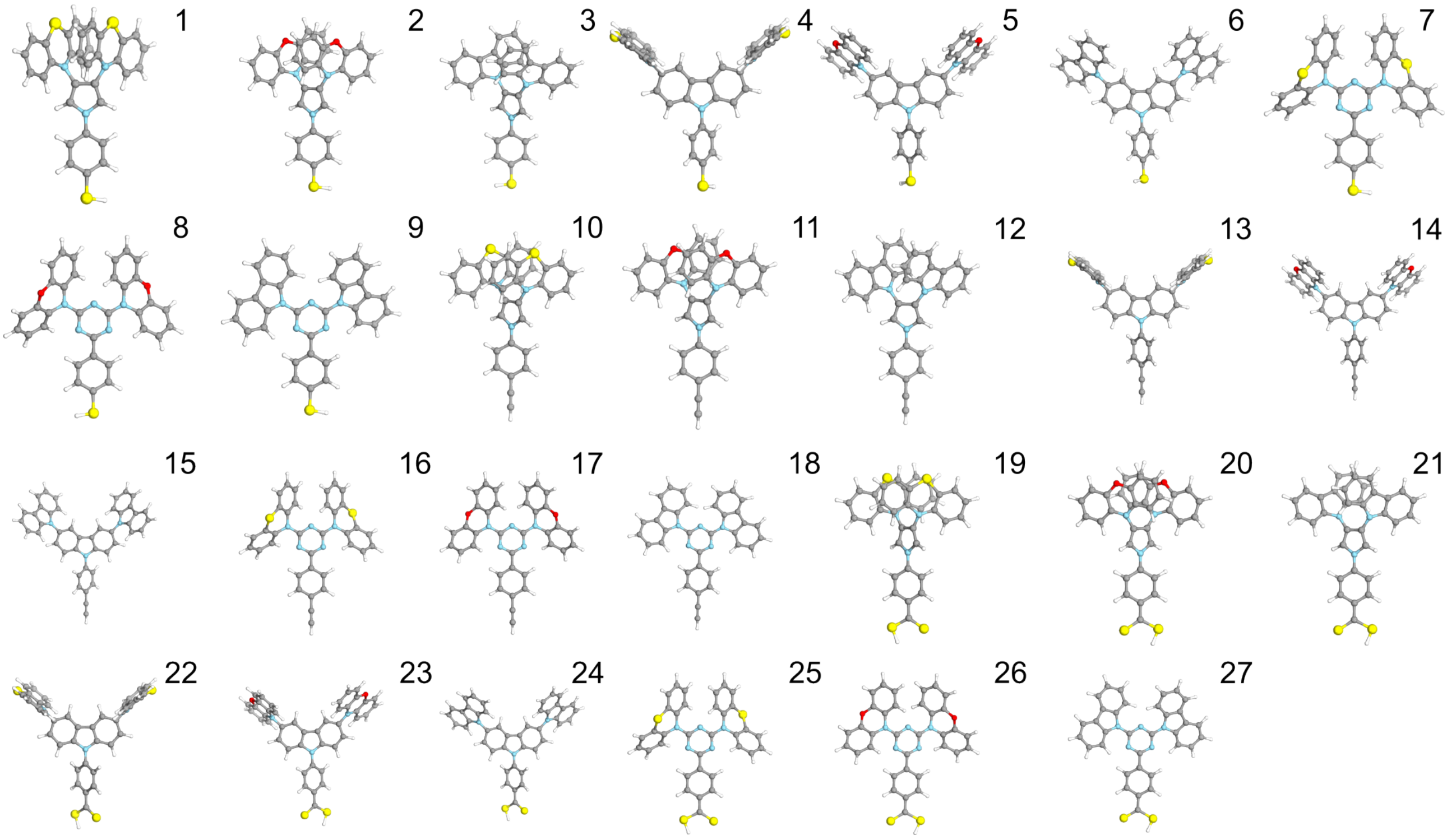


Figure S1: Lowest-energy conformers of the molecular dataset considered in this work. The initial dataset comprises 27 synthetically accessible Y-shaped molecular candidates obtained by combining different anchoring groups (AGs), spacer groups (SPs), and polarizable groups (PGs). These optimized geometries constitute the starting point for the conformational, electrostatic, and circuit-level analyses presented in the main manuscript.

## 2. Oxidized-State Electrostatic Properties

Grouping compounds by spacer identity clarifies these trends: the distance $w$ between the nitrogen atoms linking the PGs to the spacer increases from Pyr (∼3 Å) to Trz (∼4.5 Å) to Cbz (∼8 Å) (Fig. S2(a)), correlating with the observed rise in $\alpha$ (Fig. S2(b)) and the larger dispersion in both $|\mu|$ and $|\mu_y|$ (Fig. S2(c,d)) for Cbz, reflecting greater conformational flexibility. Grouping by SP–AG–PG combinations (Fig. S3) further shows that Cbz yields the largest number of conformers, Trz the fewest (consistent with higher rigidity), and CDME anchors systematically increase $\mu_x$ due to their stronger electron-withdrawing character (Fig. S4). Notably, in the Cbz–Cbz family, $\mu_x$ values are systematically lower and can even become slightly negative for Th and CAu terminations, indicating a more balanced charge distribution between the two PG branches and therefore a reduced intrinsic logical bias.

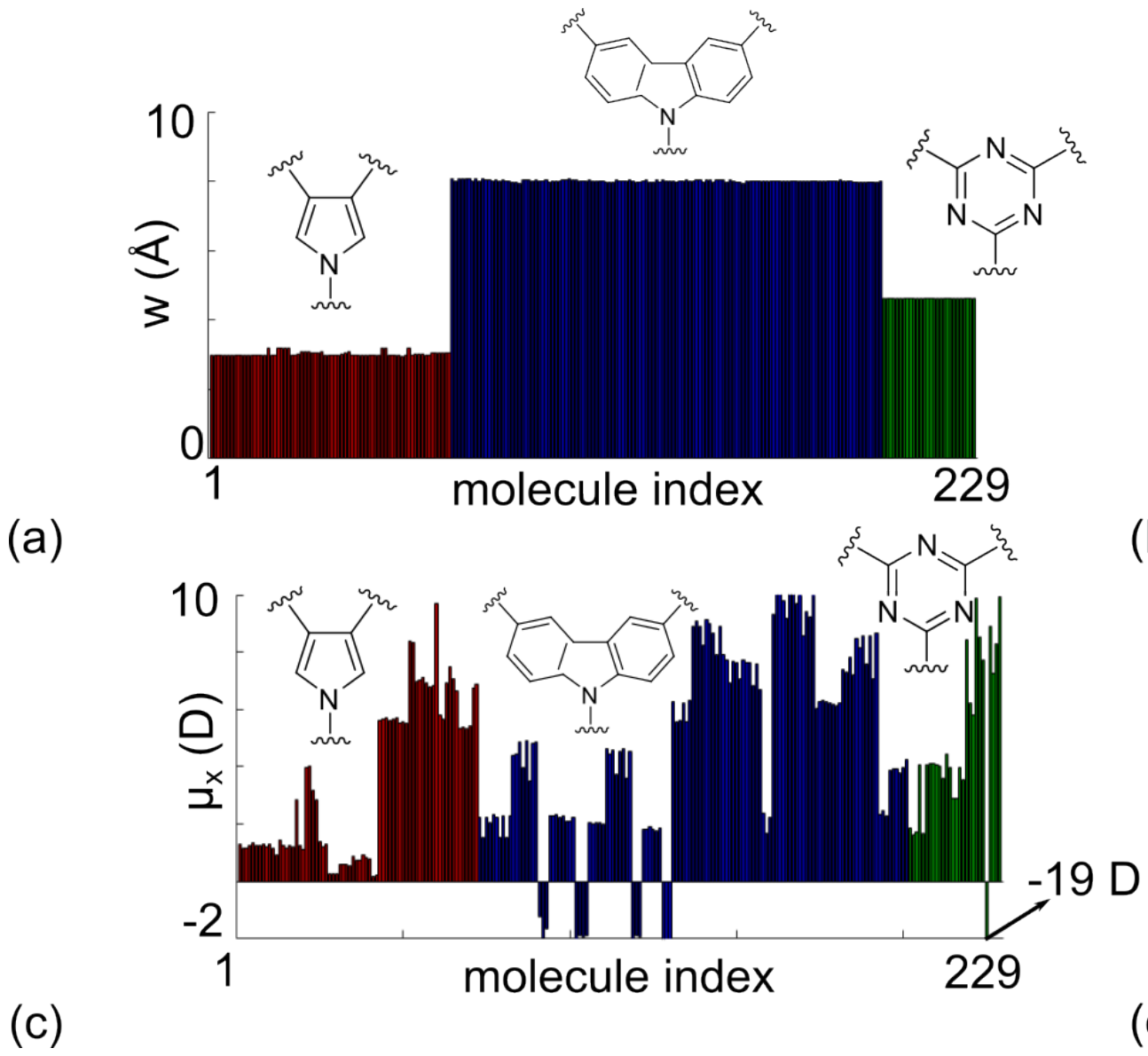


Figure S2: Structural and electrostatic descriptors of the oxidized molecular conformers, grouped according to the spacer identity: pyrrole (Pyr, red), carbazole (Cbz, blue), and triazole (Trz, green). (a) Distance $w$ between the nitrogen atoms connecting the spacer to the two polarizable groups. (b) Isotropic polarizability $\alpha$. (c) Total dipole-moment magnitude $|\mu|$. (d) Magnitude of $|\mu_y|$, which quantifies the intrinsic charge imbalance between the two logical branches. Each bar represents an individual conformer, ordered according to the molecular index.

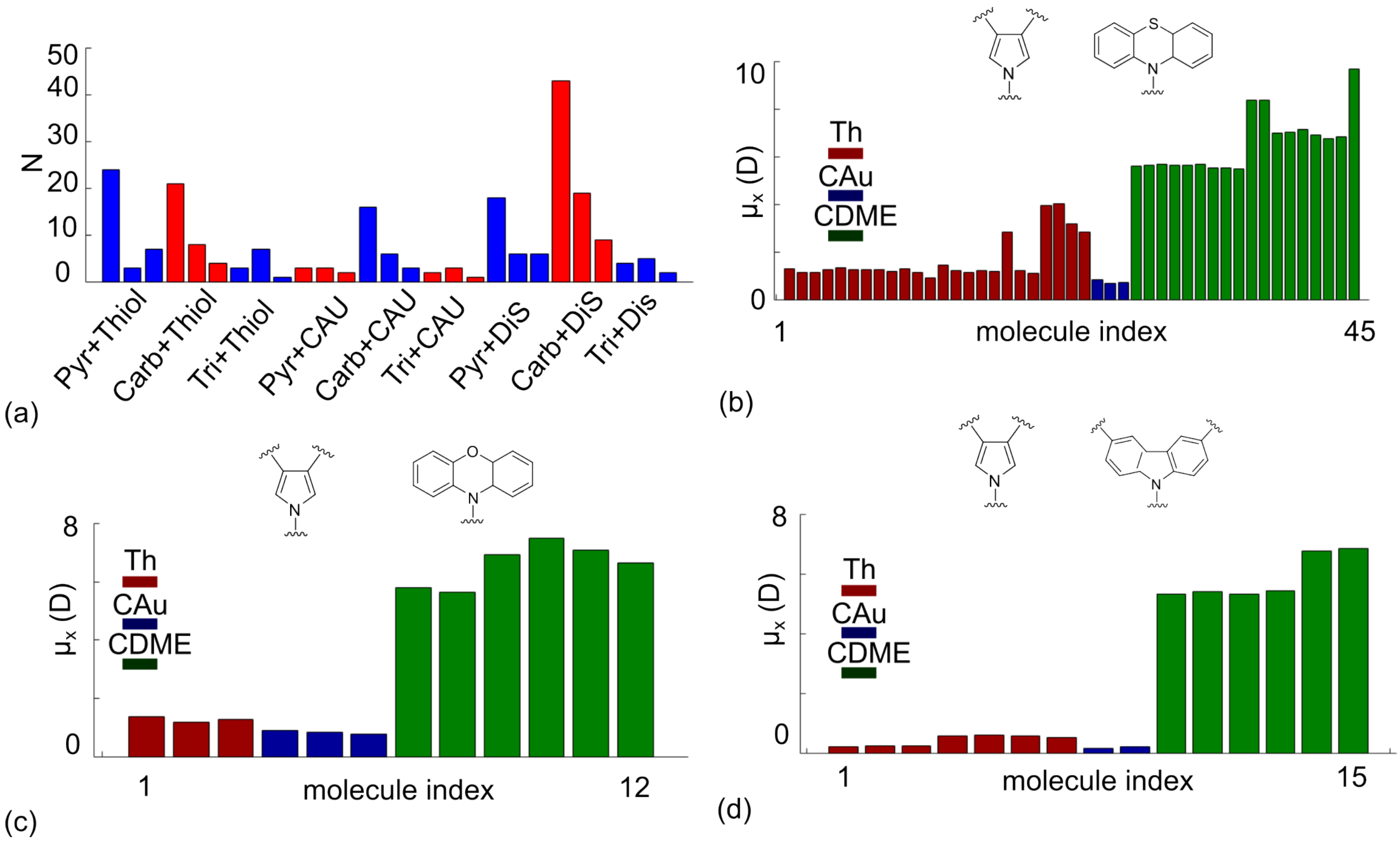


Figure S3: Influence of the anchoring group on the oxidized-state molecular response for selected AG–SP combinations. (a) Number of conformers identified for each molecular family after CREST sampling. (b–d) Dipole moment component $\mu_x$ for representative AG–SP combinations, highlighting the influence of the anchoring chemistry on the native Hold-state polarization. Red, blue, and green bars identify Th, CAu, and CDME anchoring groups, respectively, while each bar corresponds to an individual conformer.

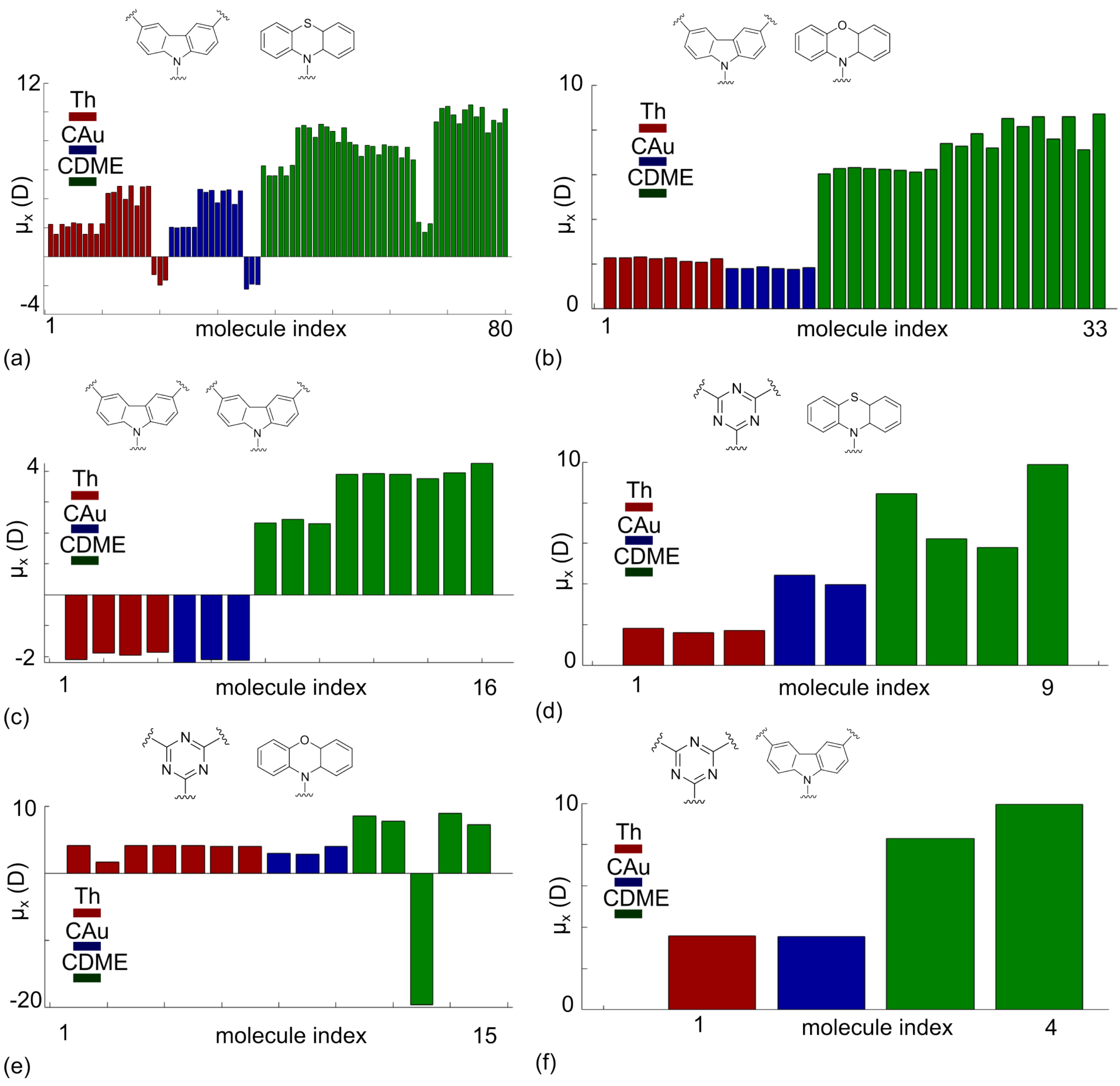


Figure S4: Oxidized-state dipole component $\mu_x$ grouped according to the anchoring group for different spacer–polarizable-group combinations. Panels (a–f) correspond to the molecular families reported above each subplot. Red, blue, and green bars identify Th, CAu, and CDME anchoring groups, respectively. The comparison illustrates how the anchoring chemistry systematically shifts the intrinsic molecular polarization while preserving the trends imposed by the molecular backbone.

## 3. Neutral-State Electrostatic Properties

Subgroup analysis (Fig. S5–S7) confirms that CDME anchors tend to increase $\mu_x$ also in the neutral state, and that Cbz–PTZ combinations show small oscillations around $\mu_x \approx 0$, while high-energy conformers of **M4** and **M13** display reduced $\mu_x$ in both charge states, indicating geometry-driven rather than oxidation-driven effects.

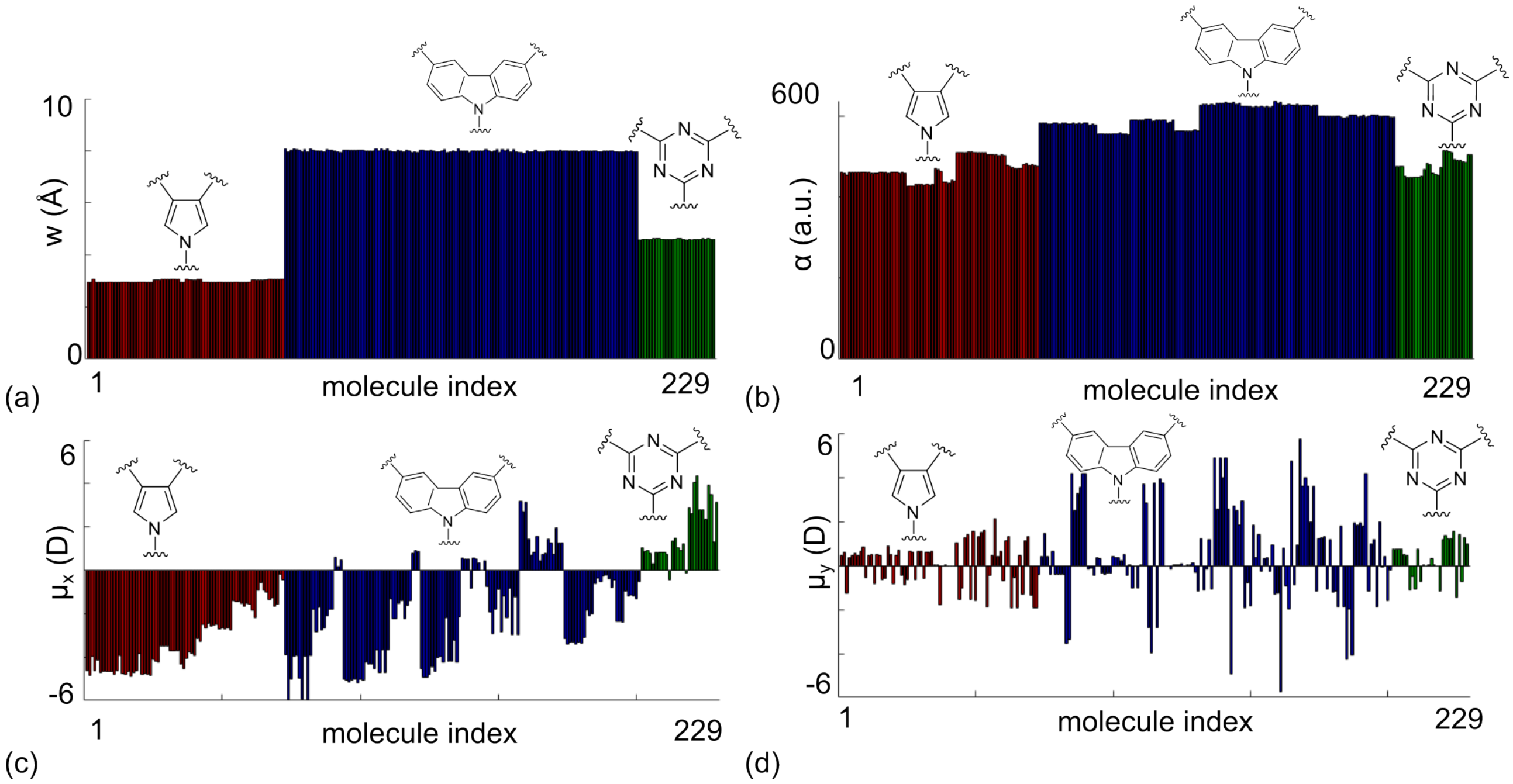


Figure S5: Structural and electrostatic descriptors of the neutral molecular conformers grouped according to the spacer identity: pyrrole (Pyr, red), carbazole (Cbz, blue), and triazole (Trz, green). (a) Distance $w$ between the nitrogen atoms connecting the spacer to the polarizable groups. (b) Isotropic polarizability $\alpha$. (c) Dipole component $\mu_x$. (d) Lateral dipole component $\mu_y$. Comparison with Fig. S2 shows that oxidation has only a minor effect on the molecular geometry and polarizability, whereas the dipole components undergo significant changes in both magnitude and sign.

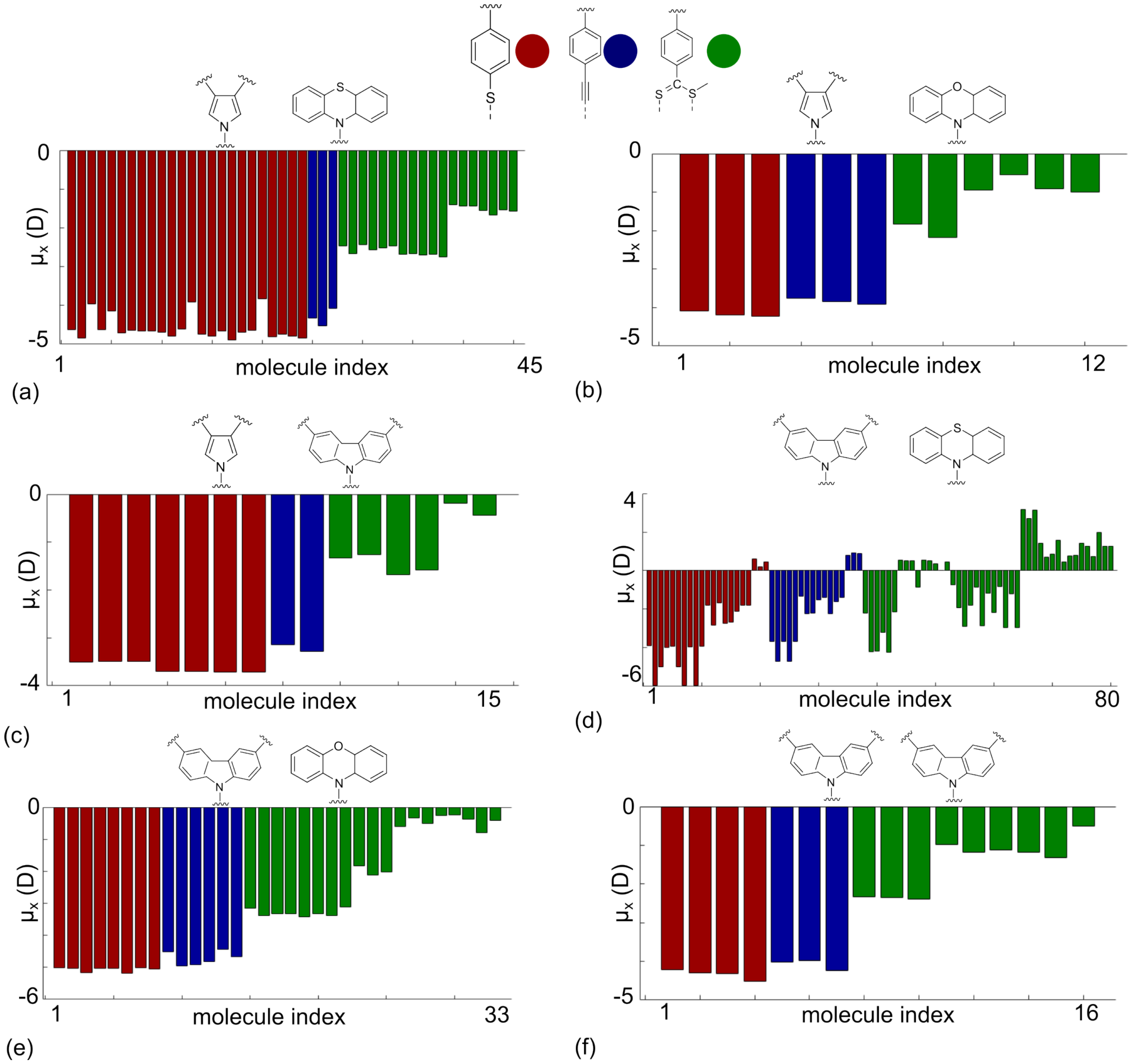


Figure S6: Neutral-state dipole component $\mu_x$ for representative PG–SP combinations. Panels (a–f) correspond to the molecular families indicated above each subplot. Red, blue, and green bars identify Th, CAu, and CDME anchoring groups, respectively. The persistence of the same subgroup trends observed in the oxidized state confirms that the molecular geometry, rather than the oxidation state, is the dominant factor controlling the intrinsic molecular polarization if the same overall oxidation state is considered (neutral or oxidized).

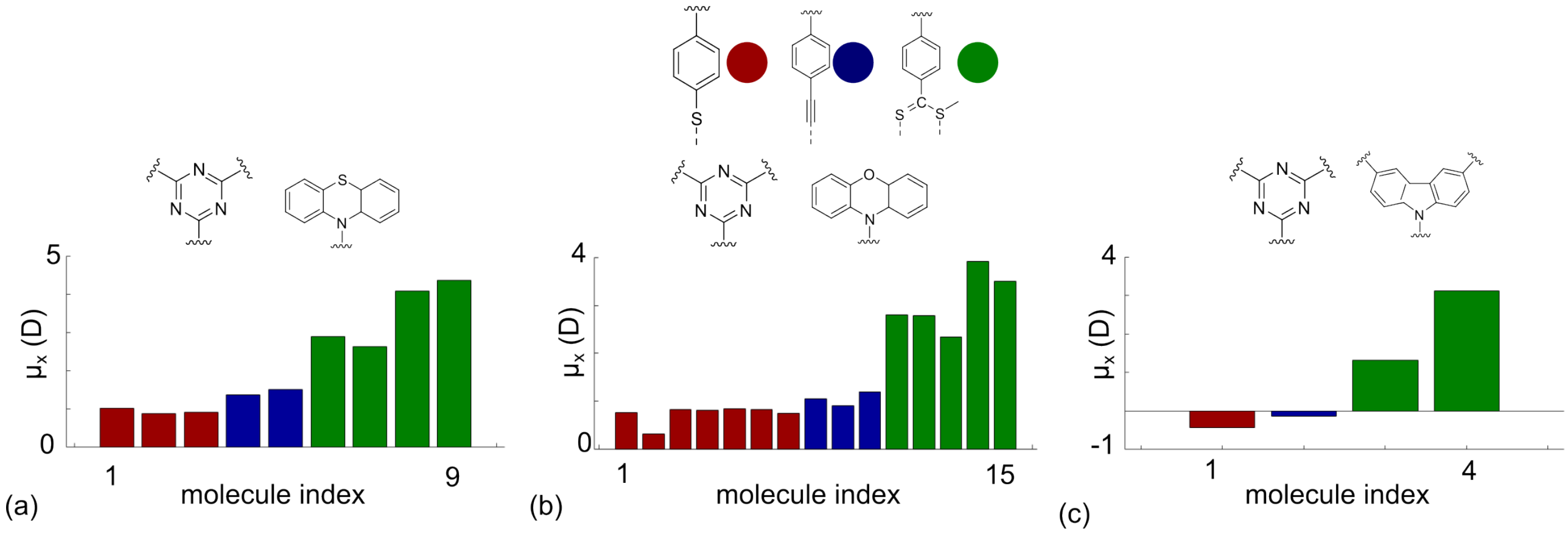


Figure S7: Neutral-state dipole component $\mu_x$ for selected Trz-linked molecular families. Panels (a–c) report different AG–PG combinations. The limited dispersion and preservation of the trends observed in the oxidized state further support the conclusion that the electrostatic response is primarily determined by the molecular geometry and conformational arrangement rather than by the oxidation state.

## 4. Phenyl-Substituted Derivatives

In the oxidized state, phenyl insertion increases $w$ (up to ~6–8 Å for Pyr and ~1.2 nm for Trz, Fig. S10(a)) and boosts $\alpha$ up to ~1000 a.u. for **M32** (Fig. S10(b)). Most derivatives show positive $\mu_x$ (Fig. S9(a)), except **M32**; Pyr-based species display low $|\mu_y|$, while Trz-linked analogues exhibit larger fluctuations. The specularity coefficient $S_y$ also increases (Fig. S9(b)), indicating flatter and less mirror-symmetric Y-geometries. Notably, Trz-based molecules cluster at well-defined $S_y$ values, reflecting their intrinsically constrained rotational freedom, whereas Pyr-based derivatives exhibit a broader $S_y$ dispersion due to increased conformational flexibility. In the neutral state, $w$ remains nearly unchanged (Fig. S11(a)), while $\alpha$ remains higher than in the corresponding non-phenyl analogues, although lower than in the oxidized state (Fig. S11(b)). The $\mu_x$ component spans positive to slightly negative regimes depending on the SP–PG combination (Fig. S9(c)), with **M31** and **M32** showing $\mu_y \approx 0$ except for a few polarized conformers. A similar specularity trend is observed in Fig. S9(d), where Trz-linked species again group into localized $S_y$ clusters, while Pyr-linked systems display a wider spread due to additional rotational degrees of freedom. Overall, phenyl insertion provides a simple and modular strategy to enhance polarizability, tune dipole symmetry, and adjust Y-geometry, thus delivering more suitable candidates for MolFCN operation.

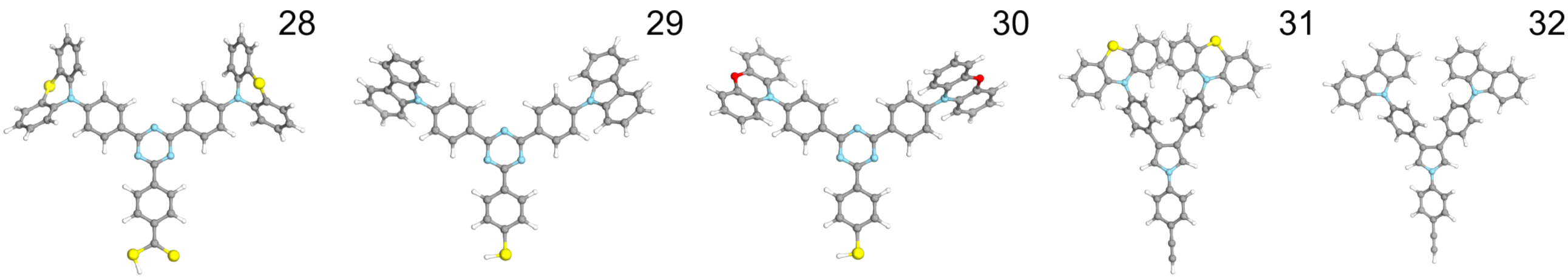


Figure S8: Phenyl-substituted molecular derivatives M28–M32 rationally designed from the systematic analysis of the initial molecular dataset. The insertion of a phenyl unit between the spacer and the polarizable groups increases their separation and provides an additional strategy to enhance molecular polarizability while preserving compatibility with the MolFCN architecture.

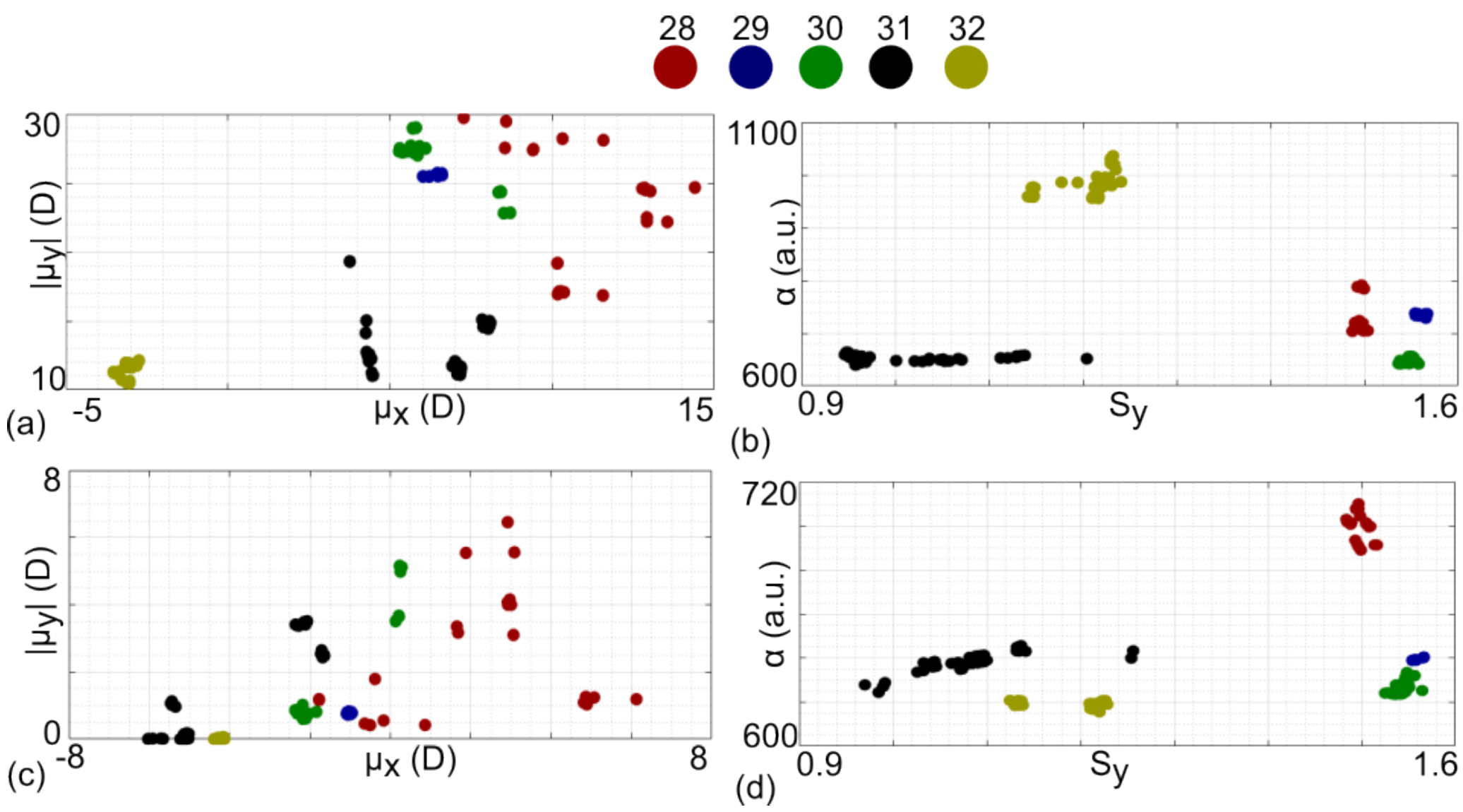


Figure S9: Electrostatic descriptors of the phenyl-substituted derivatives M28–M32. (a) Oxidized-state $\mu_x$ versus $|\mu_y|$. (b) Oxidized-state isotropic polarizability $\alpha$ versus the specularity coefficient $S_y$. (c) Neutral-state $\mu_x$ versus $|\mu_y|$. (d) Neutral-state $\alpha$ versus $S_y$. Each point corresponds to an individual conformer, while colors identify the different molecular derivatives according to the legend.

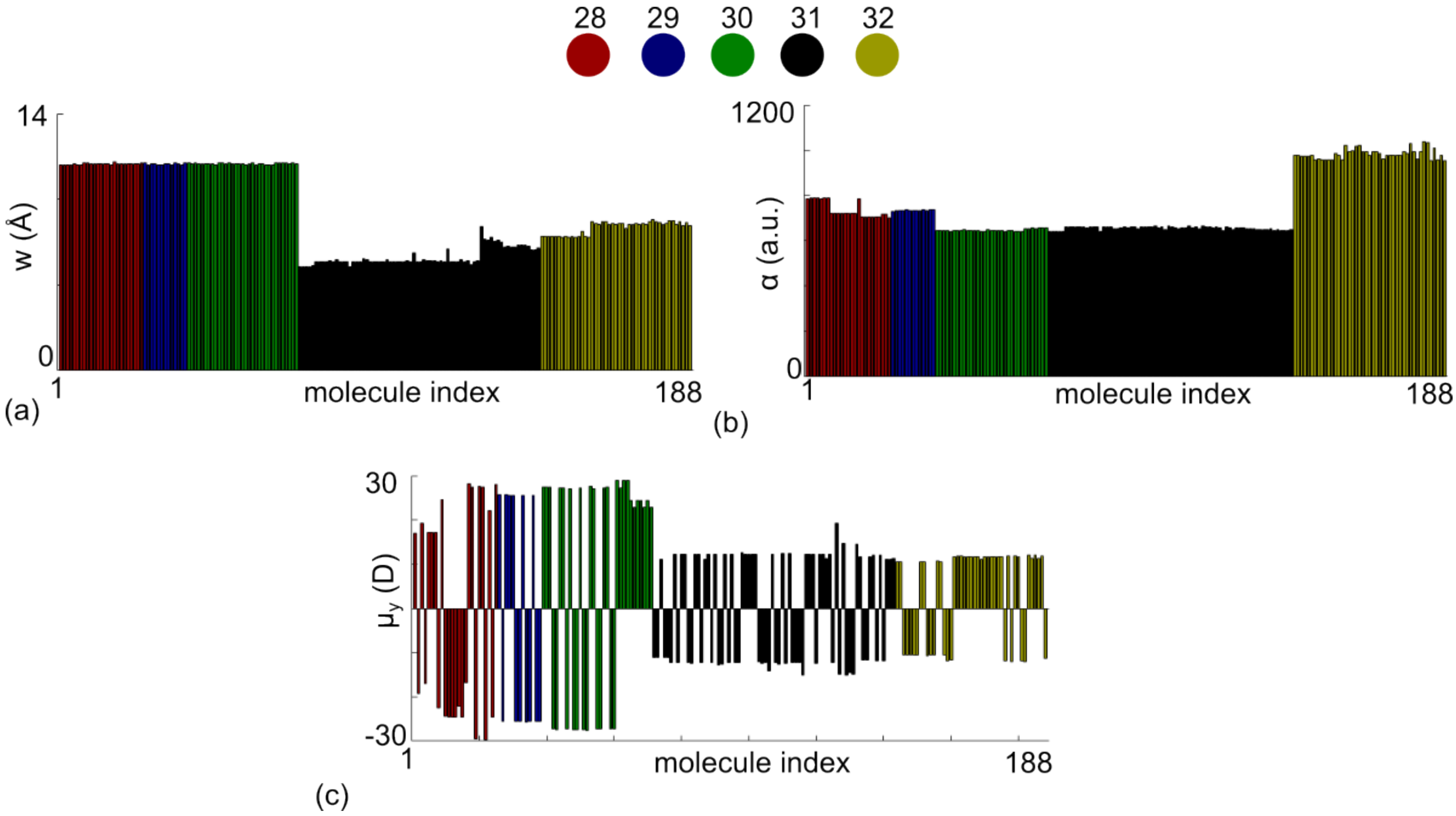


Figure S10: Structural and electrostatic properties of the oxidized phenyl-substituted derivatives. (a) Distance $w$ between the nitrogen atoms connecting the spacer to the polarizable groups. (b) Isotropic polarizability $\alpha$. (c) Lateral dipole component $\mu_y$. Each bar corresponds to an individual conformer, while colors identify molecules M28–M32 according to the legend.

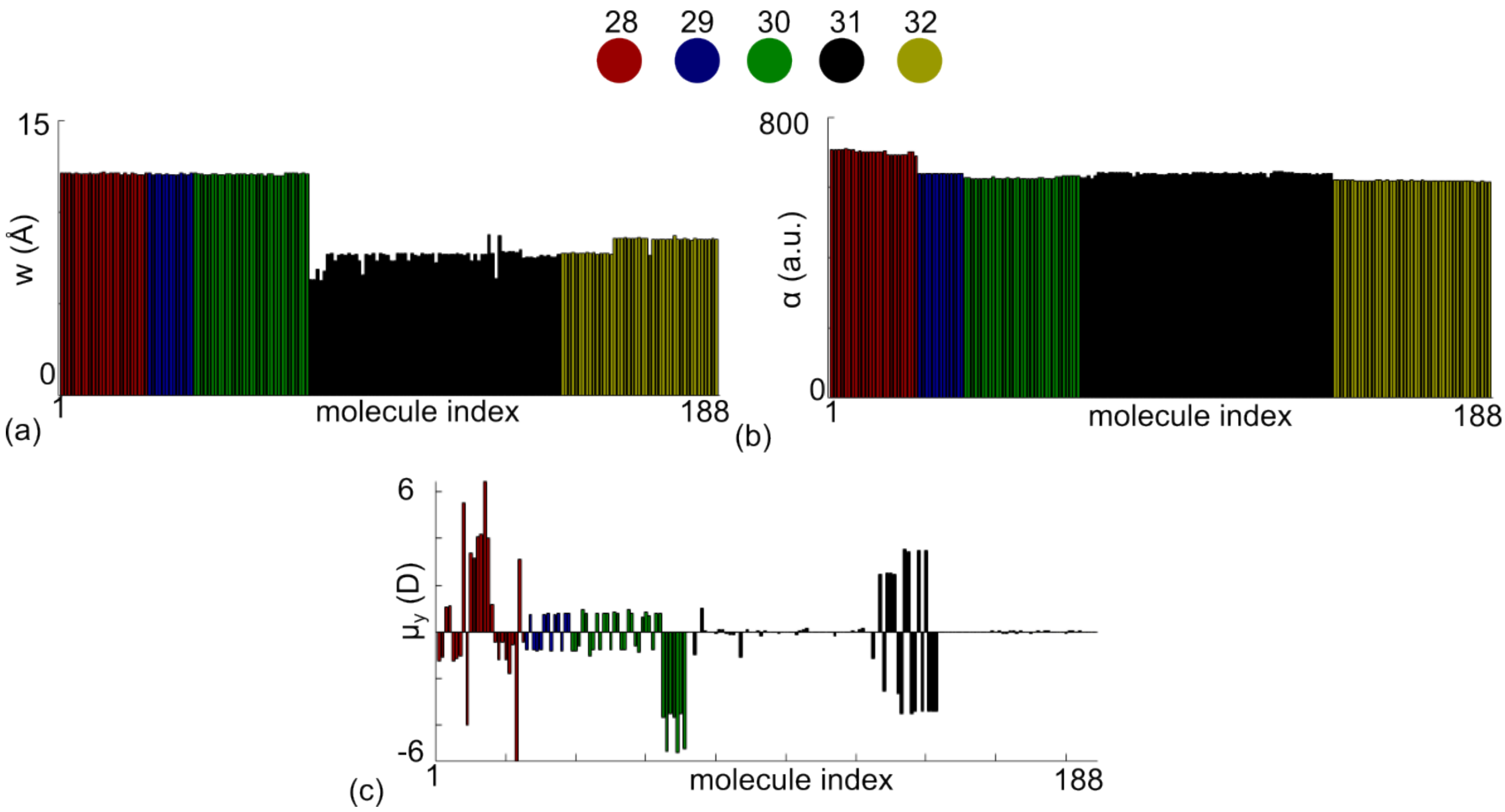


Figure S11: Structural and electrostatic properties of the neutral phenyl-substituted derivatives. (a) Distance $w$. (b) Isotropic polarizability $\alpha$. (c) Lateral dipole component $\mu_y$. Comparison with Fig. S10 shows that the structural descriptors remain largely unchanged upon oxidation, whereas the electrostatic response exhibits a reduced dipole polarization.

## 5. Semi-static molecular response and VACT validation

This section provides additional details supporting the results discussed in the main text. In particular, the influence of the semi-static treatment, the VACT extraction procedure, the perturbation-based charge grouping strategy, and the role of conformational averaging are analyzed in greater detail.

### Semi-static molecular response

To address the limitations of the static treatment, we introduce a semi-static approach in which, for each value of $V_{in}$, the molecular geometry is re-optimized under the applied $E_{ck}$, allowing for a structural response to the external fields. This methodology is applied to a representative subset of candidate species.

Figs. 3(b–c) of the main text show results for **M6**, identified as the best MolFCN candidate among the analysed compounds. The $V_{in}$–$\mu_y$ curves differ significantly between the two approaches: the static case ($\mu_s$) displays a smooth and weak dependence on the input voltage, whereas the semi-static case ($\mu_{ss}$) exhibits a sharp transition near $V_{in} = 0$ V, markedly enhancing MolFCN performance. Likewise, at $V_{in} = 0$ V, the $E_{ck}$–$\mu_{x,y}$ characteristics diverge, showing a $\sim 10$ D difference in $\mu_y$ at $E_{ck} = -2$ V/nm (Fig. 3(c)). A similar behavior is observed for **M15** (Fig. S12(a–b)), where the rotational flexibility of the Cbz–Cbz polarizable units modulates charge transfer, making the semi-static treatment essential. By contrast, **M14**, **M9**, and **M29** (Fig. S12(c–h)) show only minor differences between $\mu_s$ and $\mu_{ss}$, indicating limited geometry reorganization.

In **M14**, $\mu_x$ remains essentially unchanged even at $E_{ck} = -2$ V/nm (Fig. S12(c)), suggesting that oxygen-mediated charge stabilization prevents induction of the *Null* state. **M9** and **M29** instead achieve $\mu_y = 0$ D at $E_{ck} = -2$ V/nm, indicating proper reset behavior, similarly to **M6** and **M15**. However, **M9** does not exhibit any $\mu_y$ sign inversion across the investigated $V_{in}$ range at $E_{ck} = 0$ V/nm (Fig. S12(f)), preventing logic-state switching and making it unsuitable for MolFCN operation. In contrast, **M29**, which differs only through the addition of phenyl substituents, displays a clear $\mu_y$ sign inversion between positive and negative $V_{in}$ values (Fig. S12(h)), although with an asymmetric response corresponding to a negative switching threshold around $-0.25$ V.

Fig. S13 reports the neutral-state behavior of **M6** and **M29**, where positive $E_{ck}$ values are applied to evaluate *Hold*-state stabilization given their intrinsically negative or weak $\mu_x$. In this case, the semi-static and static treatments yield nearly identical $E_{ck}$–$\mu$ trends at $V_{in} = 0$ V, with only minor deviations at $E_{ck} = 2$ V/nm, and they overlap almost perfectly when varying $V_{in}$ at $E_{ck} = 0$ V. Since the dipole variation in the neutral state is significantly weaker than in the oxidized configuration and insufficient to sustain robust information transfer, the following analysis focuses on oxidized molecular states, previously identified as the most effective operating condition for MolFCN information propagation. Moreover, because the semi-static approach explicitly accounts for field-induced structural rearrangements and therefore provides a more realistic description of the molecular response, it is adopted throughout the subsequent discussion.

### VACT extraction and perturbation-based grouping

For the VACT extraction using the three-point-charge model in MoSQuiTo, validity requires that $\mu_{x,AC}$, $\mu_{y,AC}$, $\mu_{z,AC}$ and $V_{\text{out},AC}$ reproduce the DFT-derived dipole components ($\mu_{x,DFT}$, $\mu_{y,DFT}$, $\mu_{z,DFT}$) and output potential $V_{\text{out},DFT}$ (Fig. 3(d) of the main text). The conventional approach partitions the molecule into three geometrically defined regions and sums the atomic charges within each region to construct the aggregated charges. While straightforward, this spatial grouping remains fixed and therefore cannot accurately adapt to field-induced charge redistribution occurring under different electrostatic conditions.

To overcome this limitation, a perturbation-based grouping optimization was introduced, in which atomic charges are iteratively exchanged between dot1 and dot2 to reduce the $V_{\text{out},AC}$–$V_{\text{out},DFT}$ deviation, followed by a refinement involving dot3 (Fig. 3(e)). In addition, different molecular conformers may exhibit distinct intrinsic dipoles and VACT responses. To account for these effects, conformer-averaged VACTs are obtained through the Boltzmann-weighted averaging procedure described below

and in Eq. 2 of the main text.

These improvements were applied to representative candidates containing Cbz and Trz+Phenyl spacers, identified as the most polarizable and reliable linkers. Table 1 in the main text reports the mean dipole errors along $x$, $y$, and $z$ for the analysed species, comparing the conventional spatial grouping scheme ($e_{sp}$) with the perturbation-based grouping ($e_p$). The reported error is defined as the average deviation between the dipole moments predicted by the VACT ($\mu_{AC}$) and those obtained from DFT calculations ($\mu_{DFT}$). For each conformer, the deviation is first averaged over the sampled $V_{in}$ range and then averaged over the conformer ensemble according to

$$\text{Error} = \frac{1}{N_{\text{conf}}} \sum_{c=1}^{N_{\text{conf}}} \left( \frac{1}{N_V} \sum_{i=1}^{N_V} \left| \mu_{AC}^{(c)}(V_i) - \mu_{DFT}^{(c)}(V_i) \right| \right), \tag{1}$$

where $N_{\text{conf}}$ is the number of conformers, $N_V$ is the number of sampled $V_{in}$ values, and $\mu_{AC}^{(c)}(V_i)$ and $\mu_{DFT}^{(c)}(V_i)$ are the VACT-derived and DFT-derived dipole moments, respectively.

In the spatial grouping, atomic charges are assigned to three dots by partitioning the molecular $y$ coordinates using a fixed 2 Å threshold around the molecular center. Dot1 and dot2 correspond to the nitrogen atoms of the polarizable groups, whereas dot3 is positioned on the carbon atom of the anchoring group bound to the SP. The perturbation-based grouping uses this spatial assignment only as an initial guess and subsequently refines the grouping through charge exchanges.

As shown in Table 1 in the main text, the perturbation-based procedure consistently yields lower reconstruction errors than the conventional spatial grouping. As a consequence, $e_p$ is significantly lower than $e_{sp}$ in all investigated cases. The first-conformer errors remain below 1 D for $\mu_x$ and around 1 D for $\mu_y$, confirming the accuracy of the extracted VACTs. Although the spatial grouping could in principle be manually refined, doing so would require molecule-specific tuning and would remain unable to adapt to varying electrostatic conditions. In contrast, the perturbation-based approach performs this optimization automatically and dynamically, resulting in consistently higher accuracy without manual intervention. Fig. 3(f) illustrates the perturbation-based reconstruction accuracy for the first conformer of **M6** at $E_{ck} = -1$ V/nm, where the VACT-derived $V_{in}$–$\mu$ characteristics closely reproduce the DFT reference.

## Conformer averaging and VACT selection

Figure S14(a) reports the individual conformer $V_{in}$–$\mu$ characteristics as light solid lines together with the corresponding energy-weighted average shown as dashed lines. Because the conformers exhibit very similar energies (weights: 0.251, 0.250, 0.249, and 0.249), the averaged response almost overlaps with the individual contributions. This result confirms that a set of three aggregated charges can faithfully reproduce the electrostatic response, enabling reliable and automated VACT extraction, as reported in Fig. 3(g) of the main text.

Figs. S14(b–d) further detail the first-conformer analysis, the extracted VACT, and the DFT-derived conformer-averaged $V_{in}$–$\mu$ characteristics at $E_{ck} = 0$, $-1.5$, and $-2$ V/nm. At $E_{ck} = 0$ V/nm, the VACT closely resembles that obtained at $-1$ V/nm but exhibits a slightly stronger asymmetry around $V_{in} = 0$ V, arising from conformers with opposite-sign native $\mu_y$ values that average toward intermediate responses. This observation highlights the influence of conformational diversity on the effective MolFCN behavior.

At stronger clocking fields, charge redistribution becomes progressively dominant. Starting from $E_{ck} = -1.5$ V/nm, dot3 gradually accumulates positive charge, and at $E_{ck} = -2$ V/nm the system effectively reaches the reset condition, with $\mu_y$ almost completely suppressed and most of the positive charge localized on dot3. This behavior corresponds to a robust *Null* state, which is essential for reliable MolFCN operation.

Fig. S15 reports the $E_{ck} = 0$ V/nm results for **M15**, **M13**, **M4**, **M28**, and **M29**. The lowest-energy conformers exhibit small $e_{x,1st,p}$ and $e_{y,1st,p}$ values (Table 1), further confirming the accuracy of the perturbation-based grouping. **M15**, **M28**, and **M29** display asymmetric $V_{in}$–$\mu$ curves around $V_{in} = 0$ V due to their native dipoles, yet the dipole reverses at modest input voltages ($|V_{in}| < 0.5$ V), indicating accessible switching. In contrast, **M4** and **M13** require larger input voltages ($|V_{in}| \sim 0.6$ V),

consistent with a more stable native logic state, likely influenced by the higher electronegativity of the PTZ unit. **M28** remains particularly switchable, plausibly due to phenyl-induced charge delocalization.

Fig. S16 shows **M15** under $E_{ck} = -1$ V/nm, where the perturbation-based grouping remains valid. The semi-static energy-averaged VACT and the corresponding $V_{in}$–$\mu$ characteristic lie between those of the individual conformers, reflecting the near-symmetric switching behavior of the conformer ensemble around the transition region. As observed for **M6**, the applied field promotes partial charge localization on dot3, resulting in an intermediate operating condition between the *Hold* and *Null* states.

Across all investigated cases, the energy-averaged characteristics lie between those of the individual conformers, directly reflecting the influence of conformational diversity. Different geometries favor different native charge distributions, and their ensemble naturally produces intermediate responses. This observation raises the important question of whether the VACT should be derived from the lowest-energy conformer alone or from the conformer ensemble, since accurate information-propagation analysis depends critically on this choice.

As a practical criterion, the steady-state dipole obtained from molecular dynamics simulations, $\langle \Delta\mu \rangle_t$, is compared with the predictions of both the lowest-energy-conformer VACT and the ensemble-averaged VACT. A smaller deviation from the ensemble-averaged VACT indicates that multiple conformers are sampled under the applied $(V_{in}, E_{ck})$ conditions and therefore that the averaged representation provides the most physically meaningful description. Conversely, if the lowest-energy conformer yields the smallest deviation, the molecular dynamics remain localized around the ground-state geometry and the single-conformer VACT is considered sufficient. This criterion is adopted throughout the work to determine the most representative VACT for subsequent MolFCN simulations.

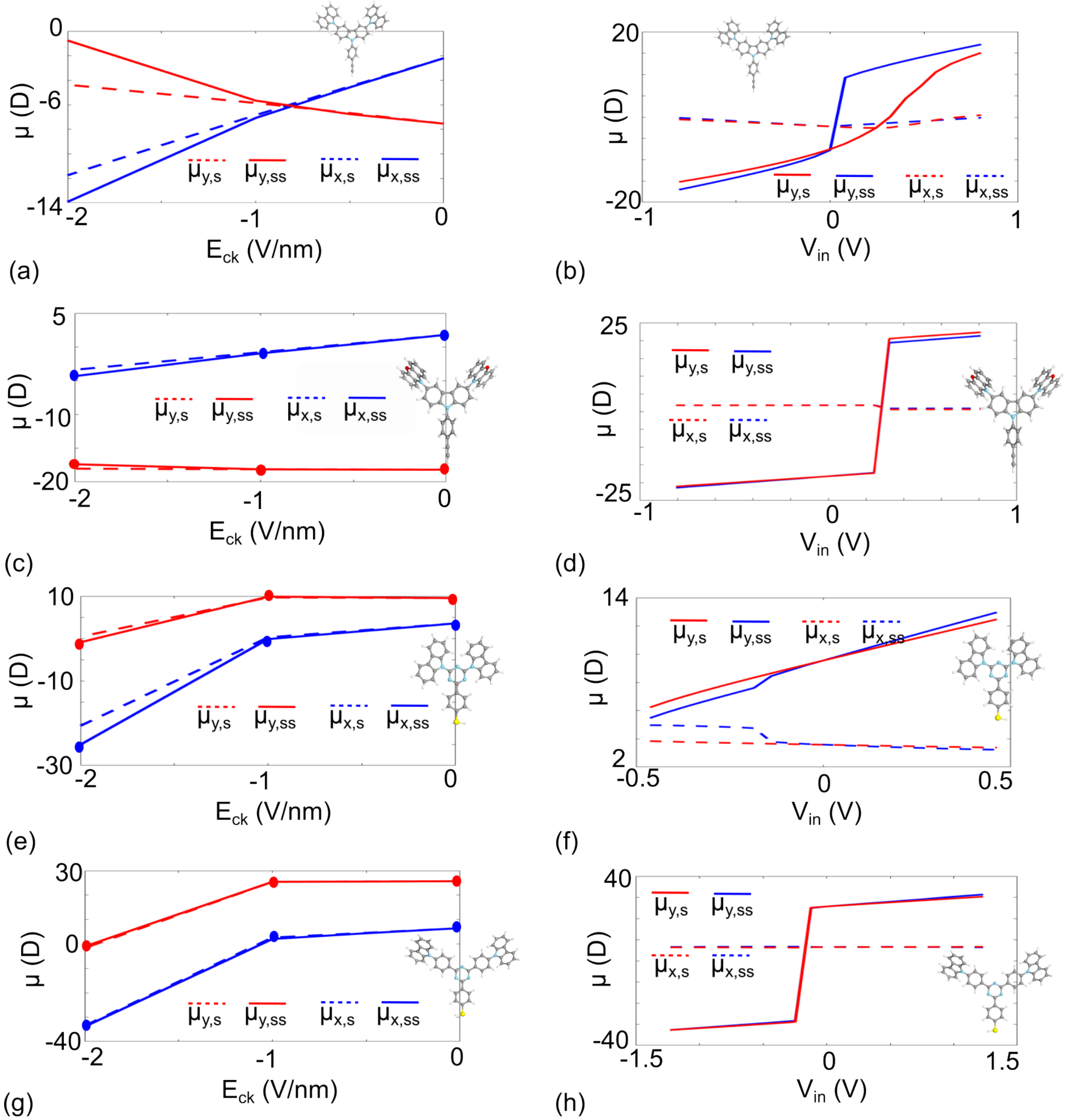


Figure S12: Comparison between the conventional static and the proposed semi-static electrostatic analyses for representative oxidized molecular candidates. The four rows correspond to molecules M15, M14, M9, and M29, respectively. Panels (a,c,e,g) compare the clock-field response ($E_{\mathrm{ck}}$–$\mu$) at $V_{\mathrm{in}} = 0$, while panels (b,d,f,h) compare the corresponding $V_{\mathrm{in}}$–$\mu$ characteristics. The semi-static treatment accounts for field-induced structural relaxation, resulting in sharper switching transitions and improved representation of the molecular electrostatic response for highly polarizable candidates.

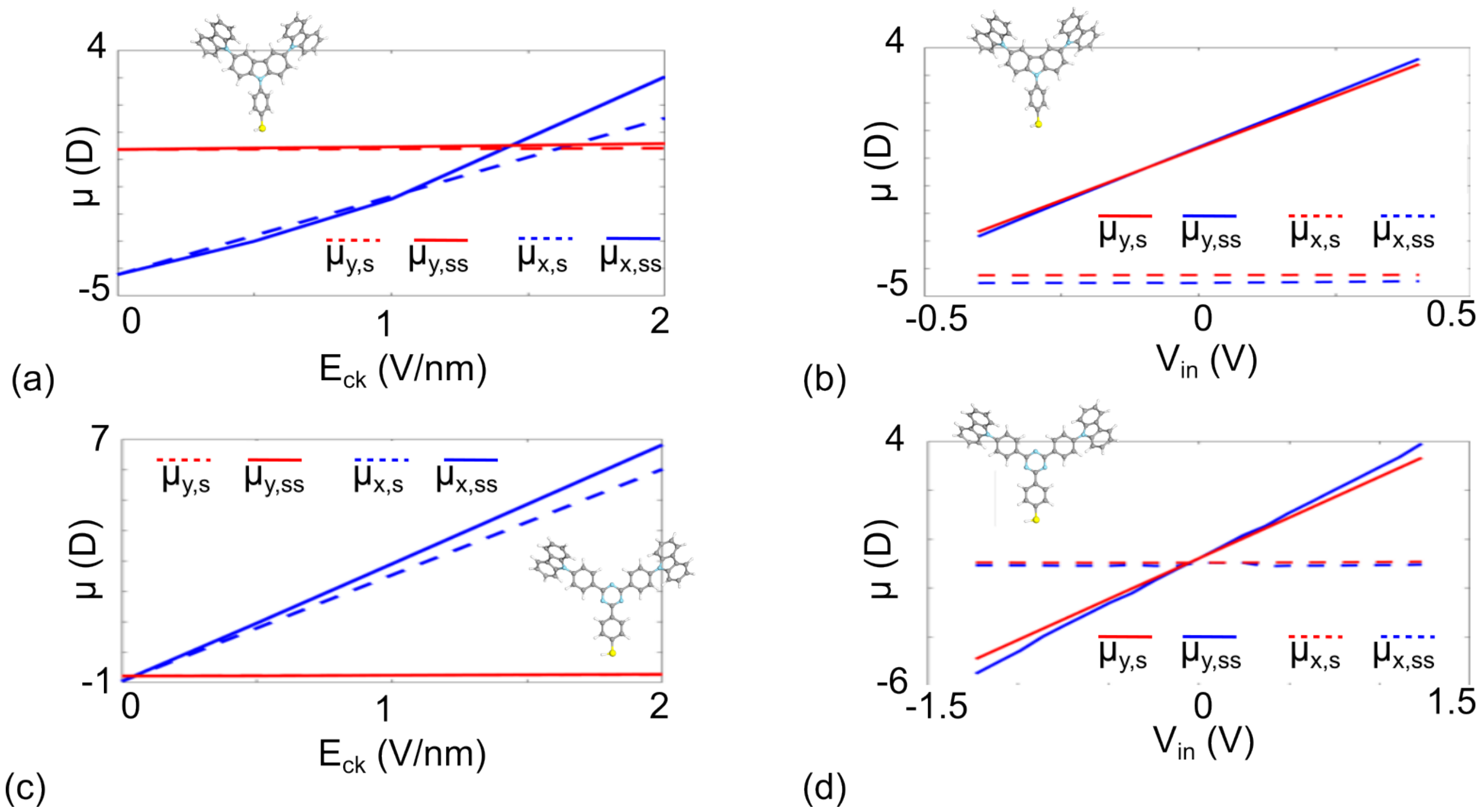


Figure S13: Comparison between static and semi-static electrostatic analyses for representative neutral molecular candidates. The upper and lower rows correspond to molecules M6 and M29, respectively. The left panels report the clock-field response ($E_{\mathrm{ck}}$–$\mu$), whereas the right panels compare the corresponding $V_{\mathrm{in}}$–$\mu$ characteristics. In agreement with the oxidized-state analysis, molecules exhibiting limited structural flexibility show only minor deviations between the two approaches.

## 6. Specularity Coefficient

To quantify molecular reflection symmetry, we define the specularity coefficient $S$. Given atomic coordinates $\mathbf{R} = \{\vec{r}_i\}_{i=1}^{N}$, the geometric center of mass is:

$$\vec{r}_{\mathrm{COM}} = \frac{1}{N}\sum_{i=1}^{N}\vec{r}_i$$

The radius of gyration is:

$$R_g = \frac{1}{N}\sum_{i=1}^{N}\|\vec{r}_i - \vec{r}_{\mathrm{COM}}\|$$

Coordinates are reflected along the Cartesian axes:

$$\begin{aligned} \vec{r}_i^{(x)} &= (-x_i,\ y_i,\ z_i), \\ \vec{r}_i^{(y)} &= (x_i,\ -y_i,\ z_i), \\ \vec{r}_i^{(z)} &= (x_i,\ y_i,\ -z_i) \end{aligned} \tag{2}$$

The mean deviations are:

$$\begin{aligned} S_x &= \frac{1}{N}\sum_{i=1}^{N}\left\|\vec{r}_i - \vec{r}_i^{(x)}\right\|, \\ S_y &= \frac{1}{N}\sum_{i=1}^{N}\left\|\vec{r}_i - \vec{r}_i^{(y)}\right\|, \\ S_z &= \frac{1}{N}\sum_{i=1}^{N}\left\|\vec{r}_i - \vec{r}_i^{(z)}\right\| \end{aligned} \tag{3}$$

Normalized specularity coefficients are:

$$\tilde{S}_x = \frac{S_x}{R_g}, \quad \tilde{S}_y = \frac{S_y}{R_g}, \quad \tilde{S}_z = \frac{S_z}{R_g}$$

Finally, the global specularity coefficient is:

$$\tilde{S}_{\mathrm{global}} = \frac{1}{3}\left(\tilde{S}_x + \tilde{S}_y + \tilde{S}_z\right)$$

Lower $\tilde{S}$ values indicate higher specularity. In the main text (Fig. 2e, Fig. 2f), we report the correlation between $\alpha$ and $\tilde{S}_y$, showing clear separation of Pyr-, Trz-, and Cbz-linked molecules.

# 7. Perturbation-Based Grouping Model

In the aggregated-charge framework, the molecule is mapped onto a reduced three-point charge system, where atomic charges are partitioned into three groups. The resulting model is considered reliable only if it can reproduce the DFT reference values for the dipole moment components ($\mu_x$, $\mu_y$, $\mu_z$) and for the output potential $V_{\text{out}}$ at different $V_{in}$ values under a fixed $E_{ck}$. Conventional spatial-based grouping is, however, too rigid: it does not adapt to changes in the external fields and fails to capture the full electrostatic response of the system. To address this issue, we employ a perturbation-based optimization strategy. The method starts from an initial grouping of the atomic charges and evaluates the error between the AC- and DFT-derived $V_{\text{out}}$. Random exchanges of atomic charges between dots 1 and 2 are then performed iteratively on a predefined number of iterations. A new configuration is accepted only if the resulting error decreases compared to the previous step. After a maximum number of iterations, the configuration with the lowest error is retained as the reference for further optimization. This procedure is summarized in Algorithm 1. In the subsequent refinement stage, charge exchanges are extended to include dot 3, in order to optimize the out-of-plane contribution ($\mu_x$). At each iteration, the updated configuration is accepted only if it reduces the discrepancy in dipole components while keeping the $V_{\text{out}}$ error below a predefined threshold. This cascade optimization procedure is summarized in Algorithm 2.

The full perturbation-based algorithm has been applied to all conformers of the molecules considered in this work, ensuring that each conformer is associated with a consistent and optimized set of three point charges. These groupings were then used for VACT extraction.

**Algorithm 1** Perturbation method — Step 1 ($V_{\text{out}}$ pre-optimization)

Compute baseline error $error_{\text{last}} \leftarrow \text{Err}(V_{\text{out,AC}}, V_{\text{out,DFT}})$
**repeat**
  Propose new grouping by exchanging two random atomic charges between dots 1 and 2
  Recompute $V_{\text{out,AC}}$ and set $error_{\text{new}} \leftarrow \text{Err}(V_{\text{out,AC}}, V_{\text{out,DFT}})$
  **if** $error_{\text{new}} < error_{\text{last}}$ **then**
    Accept new configuration
    $error_{\text{last}} \leftarrow error_{\text{new}}$
  **else**
    Reject and revert to previous configuration
  **end if**
**until** maximum iterations reached

**Algorithm 2** Perturbation method — Steps 2/3/4 (cascade dipole optimization)

Set current configuration to best from Step 1
Compute baseline dipole error $error_{\text{last}} \leftarrow \text{Err}([\mu_x, \mu_y, \mu_z]_{\text{AC}}, [\mu_x, \mu_y, \mu_z]_{\text{DFT}})$
Define $V_{\text{out}}$ tolerance $\tau_{V_{\text{out}}}$
**repeat**
  Propose new grouping exchanging charges among dots 1, 2, 3
  Recompute $([\mu_x, \mu_y, \mu_z]_{\text{AC}}, V_{\text{out,AC}})$
  $error_{\text{dip}} \leftarrow \text{Err}([\mu_x, \mu_y, \mu_z]_{\text{AC}}, [\mu_x, \mu_y, \mu_z]_{\text{DFT}})$
  $error_V \leftarrow \text{Err}(V_{\text{out,AC}}, V_{\text{out,DFT}})$
  **if** $error_{\text{dip}} < error_{\text{last}}$ **and** $error_V \leq \tau_{V_{\text{out}}}$ **then**
    Accept new configuration
    $error_{\text{last}} \leftarrow error_{\text{dip}}$
  **else**
    Reject and revert to previous configuration
  **end if**
**until** maximum iterations reached

# 8. Averaging VACTs

As discussed in the main text, conformational analysis reveals that the same molecule may present markedly different dipole moments depending on the conformer, leading to different transcharacteristics. Selecting only one conformer for SCERPA simulations would neglect conformational diversity. To address this, we introduce the concept of averaged VACTs, where the effective aggregated charges are computed as the Boltzmann-weighted average over all conformers.

Formally, the average charge for each dot $j$ is:

$$\langle AC_{\text{dot j}} \rangle = \sum_i w_i \cdot AC_{\text{dot j},i}, \quad j = [1, 2, 3], , i = [1, N_{\text{conf}}] \tag{4}$$

The weights $w_i$ are derived from the single-point energies $E_i$ of each conformer as:

$$w_i = \frac{e^{-\Delta E_i / kT}}{\sum_j e^{-\Delta E_j / kT}} \tag{5}$$

where $\Delta E_i$ is the relative energy of conformer $i$, shifted with respect to the minimum energy conformer:

$$\Delta E_i = E_i - \min_j (E_j) \tag{6}$$

This approach guarantees that lower-energy conformers dominate the average while higher-energy conformers still contribute proportionally to their thermodynamic relevance. To the best of our knowledge, this represents the first MolFCN modeling explicitly accounting for the role of molecular conformers.

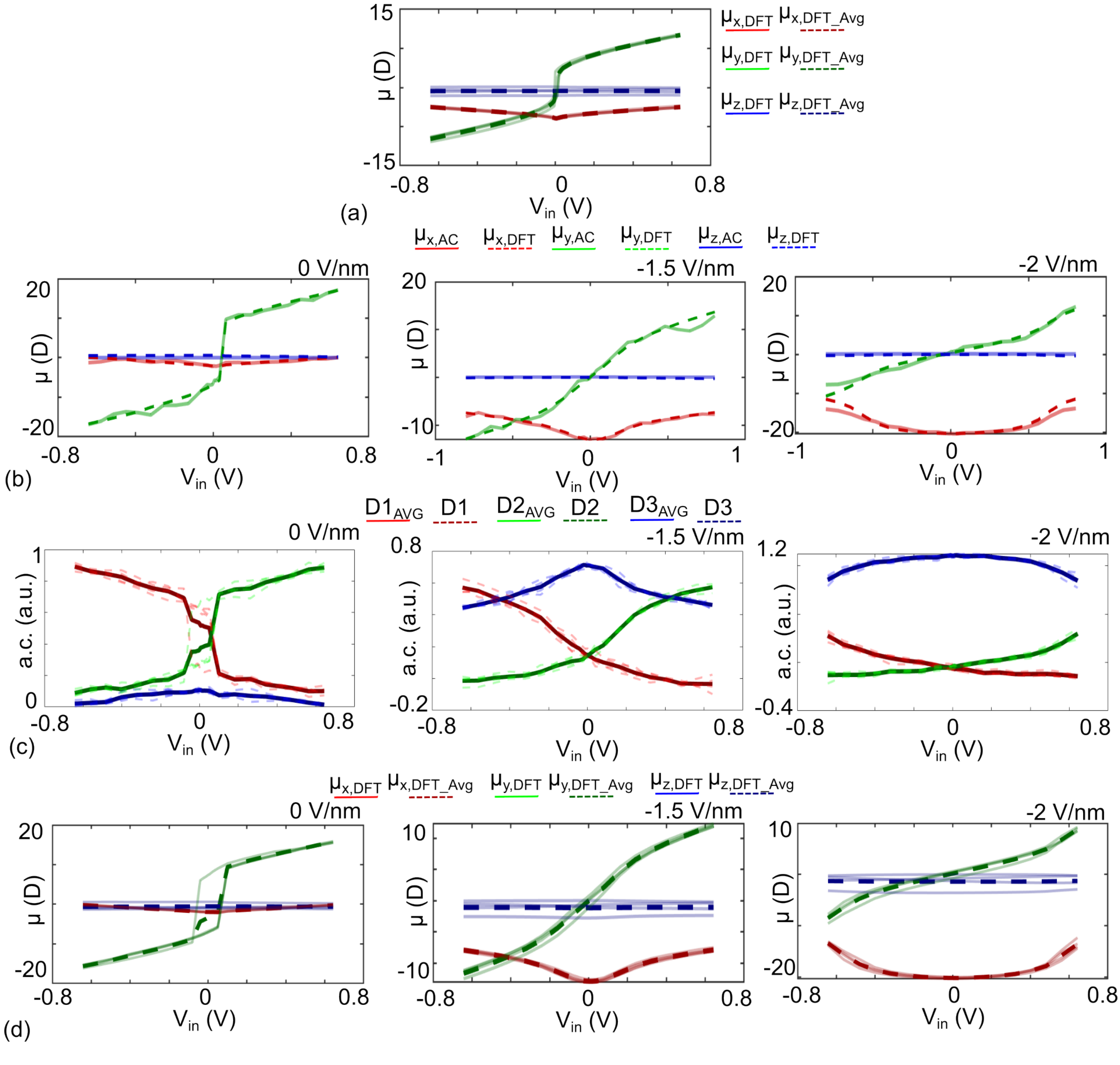


Figure S14: Validation of the conformer-averaged VACT for molecule M6. (a) Comparison between the DFT $V_{\rm in}$–$\mu$ characteristics of the individual conformers and their Boltzmann-weighted conformational average at $E_{\rm ck} = -1$ V/nm. (b) Boltzmann-weighted DFT $V_{\rm in}$–$\mu$ characteristics at $E_{\rm ck} = 0$, $-1.5$, and $-2$ V/nm. (c) Corresponding voltage-to-aggregated-charge transcharacteristics for the three effective dots. (d) Comparison between the dipole components reconstructed from the conformer-averaged VACT and the corresponding Boltzmann-weighted DFT characteristics, demonstrating the accuracy of the coarse-grained representation over the investigated operating conditions.

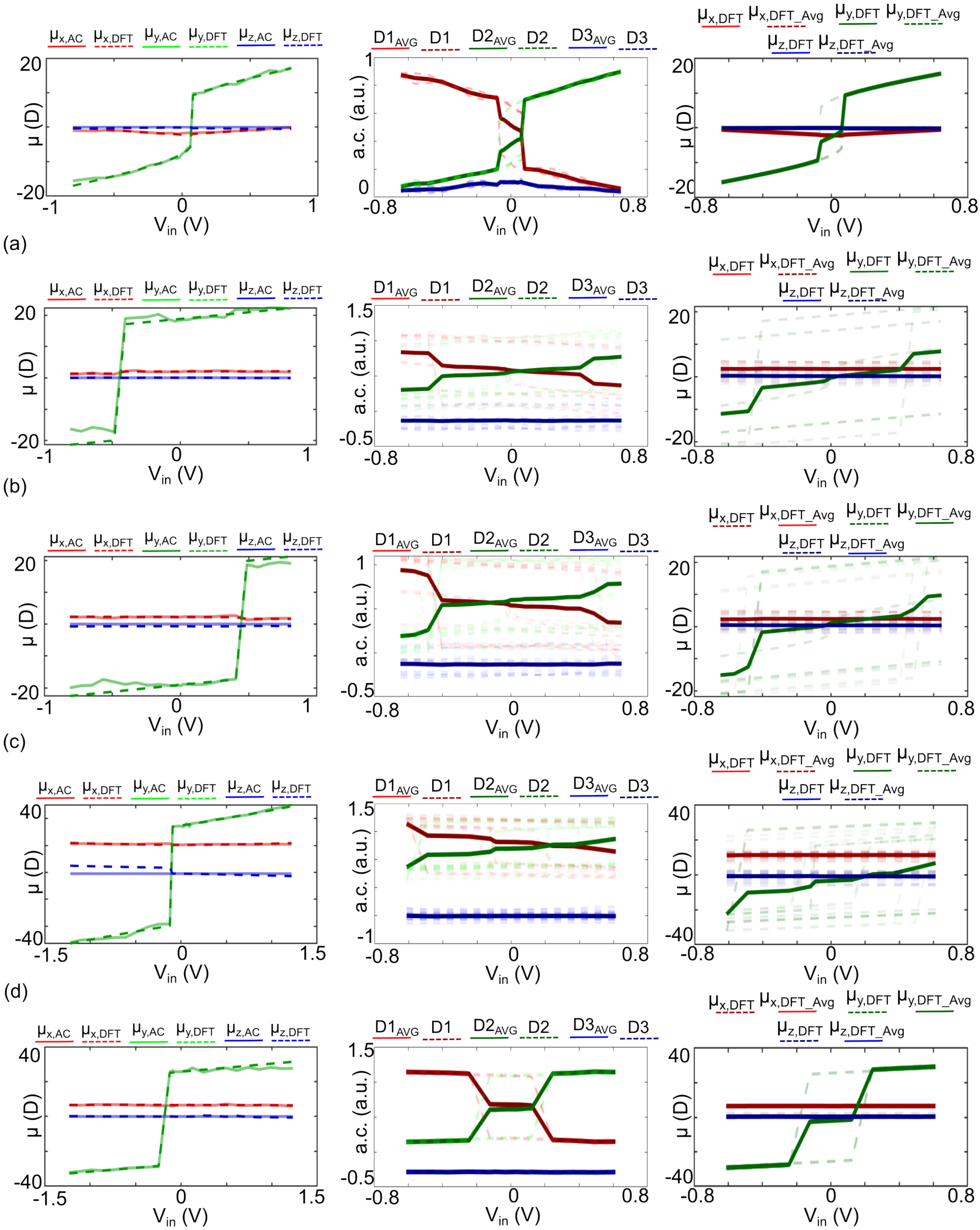


Figure S15: Validation of the conformer-averaged molecular representation for representative candidates M15, M13, M4, M28, and M29 (from top to bottom) at $E_{\mathrm{ck}} = 0$ V/nm. For each molecule, the left column compares the $V_{\mathrm{in}}$–$\mu$ characteristics of the lowest-energy conformer with the Boltzmann-weighted conformational average obtained from DFT calculations. The central column reports the corresponding aggregated-charge (AC) transcharacteristics for the three effective dots (D1–D3), comparing the lowest-energy-conformer and conformer-averaged models. The right column compares the dipole response reconstructed from the conformer-averaged AC model with the Boltzmann-weighted DFT characteristics, while the faint curves represent the individual conformers. The results demonstrate that the proposed conformer-averaged representation accurately reproduces the electrostatic response over a broad range of molecular candidates with different conformational flexibility.

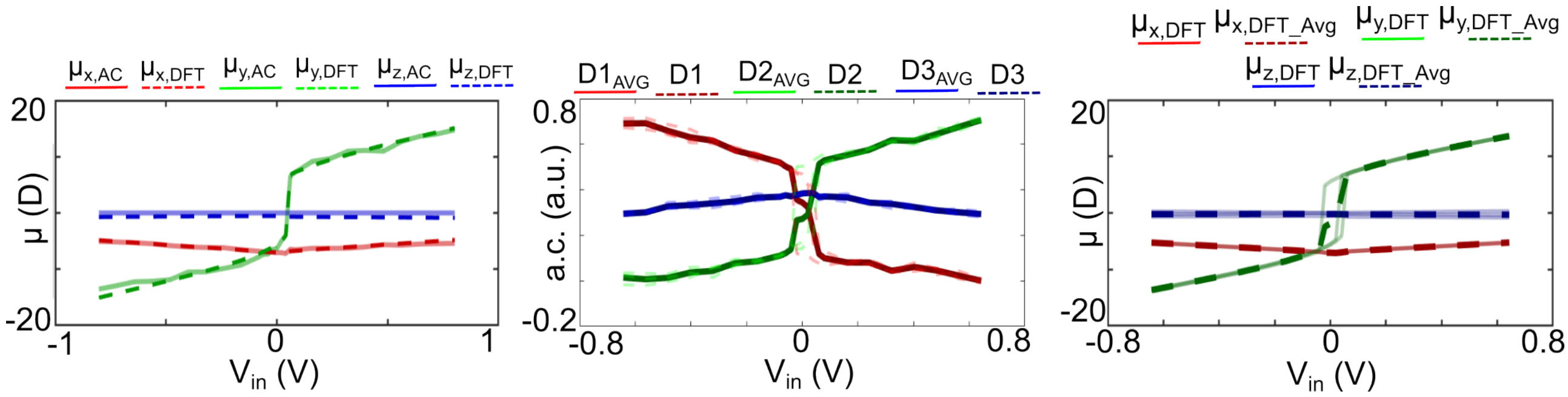


Figure S16: Validation of the conformer-averaged molecular representation for molecule M15 under an applied clock field of $E_{\mathrm{ck}} = -1$ V/nm. The left panel compares the DFT $V_{\mathrm{in}}$–$\mu$ transcharacteristics of the lowest-energy conformer with the corresponding Boltzmann-weighted average. The central panel reports the associated aggregated-charge (AC) transcharacteristics for the three effective dots (D1–D3). The right panel compares the dipole characteristics reconstructed from the conformer-averaged AC model with the corresponding Boltzmann-weighted DFT response, confirming that the averaged representation accurately reproduces the electrostatic response also under clocked operating conditions.

## 9. AIMD Validation

Additional details on the comparison between AIMD-derived dipoles and VACT predictions for **M6** are reported in this section. To complement the discussion of Fig. 4 of the main text, we present the time evolution of the dipole for the three applied biases, see Figure S17. For $E_{ck} = -1$ V/nm (Fig. 4(a)), the discrepancies at the three bias points are similar for both the energy-averaged ($\epsilon_{\text{avg}}$) and lowest-energy conformer ($\epsilon_{\text{1st}}$) predictions, with the ensemble-averaged curve overall aligning well with the AIMD time averages. The dipole dynamics show that at $V_{in} = +0.32$ V the dipole reaches steady state only after $\sim$ 100 fs (Fig. S17(c)), unlike the faster stabilization at $V_{in} = 0$ and $-0.32$ V (Fig. S17(a),(b)), because the native $\mu_y$ initially opposes the field-induced dipole and the geometry must reorganize before settling. A similar delayed settling is observed at $E_{ck} = -1.5$ V/nm (Fig. 4(b)), where the corresponding $\mu(t)$ traces (Fig. S17(d)–(f)) again show transient relaxation at $V_{in} = +0.32$ V. These transient-response behaviors directly inform time-domain MolFCN modeling, providing the limits of the intrinsic switching speed. For $E_{ck} = 0$ V/nm (Fig. 4(c)), a larger deviation appears in $\mu_y$ at $V_{in} = 0$ V, with $\epsilon_{y,\text{avg}}$ and $\epsilon_{y,\text{1st}}$ nearly identical and both exceeding 1 D. At $V_{in} = \pm 0.32$ V, the errors from both references become comparable as the system enters a saturation regime where the semi-static $V_{in}$–$\mu$ curves converge toward the AIMD averages (Fig. S17(g)–(i)). For $E_{\text{ck}} = -2$ V/nm (Fig. 4(d)), the strong clock field drives the molecule toward the *Null* configuration. At $V_{\text{in}} = 0$ V, $\mu_x$ reaches its steady-state value only after approximately 150 fs (Fig. S17(l)), while $\mu_y$ remains largely quenched. Comparable settling behavior is observed at $V_{\text{in}} = \pm 0.32$ V (Fig. S17(m),(n)), confirming that finite molecular-relaxation times must be considered when estimating the applicable clocking frequency.

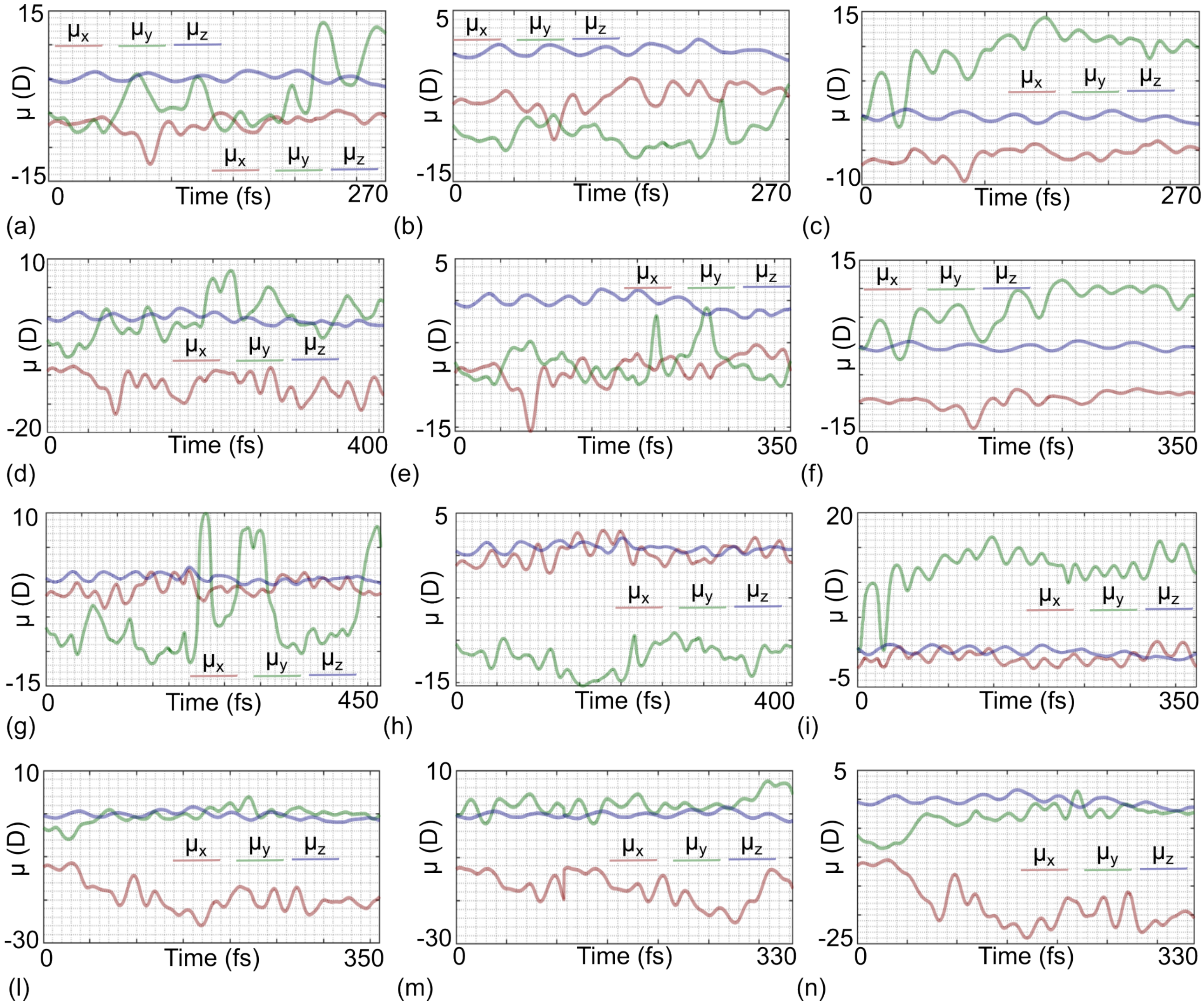


Figure S17: Time evolution of the molecular dipole components obtained from AIMD simulations for molecule M6 under different combinations of clock field and input bias. The four rows correspond to $E_{\mathrm{ck}} = -1$, $-1.5$, $0$, and $-2$ V/nm, while the three columns report $V_{\mathrm{in}} = 0.0$, $-0.32$, and $+0.32$ V, respectively. The transient dipole dynamics provide the physical basis for interpreting the agreement between the AIMD time-averaged dipoles and the semi-static VACT predictions reported in Fig. 4, highlighting the different relaxation behaviors responsible for the observed prediction errors under the various operating conditions.

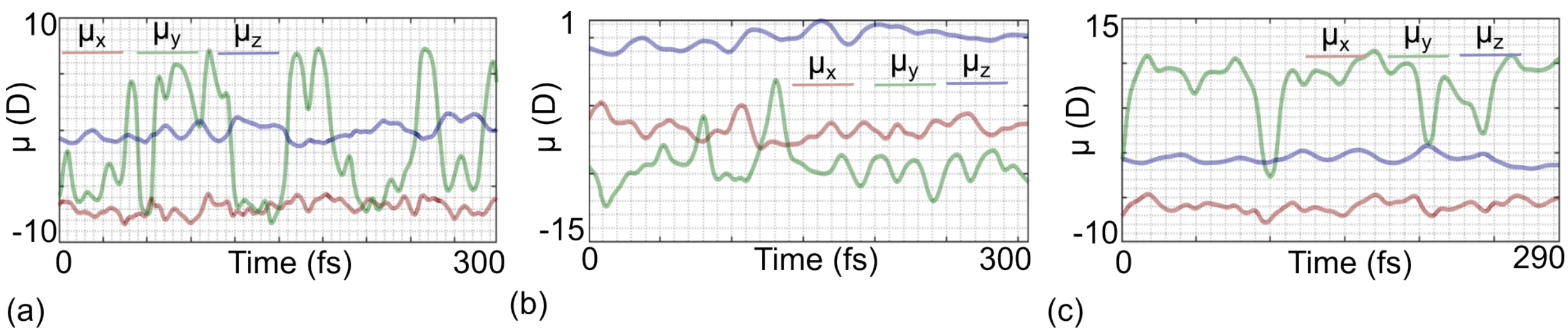


Figure S18: Time evolution of the molecular dipole components obtained from AIMD simulations for molecule M15 at $E_{\mathrm{ck}} = -1$ V/nm under three input-bias conditions: (a) $V_{\mathrm{in}} = 0.0$ V, (b) $V_{\mathrm{in}} = -0.32$ V, and (c) $V_{\mathrm{in}} = +0.32$ V. The AIMD trajectories provide the time-domain reference used to compare the time-averaged dipoles with the semi-static VACT predictions, further validating the conformer-averaged molecular representation for this molecular candidate.

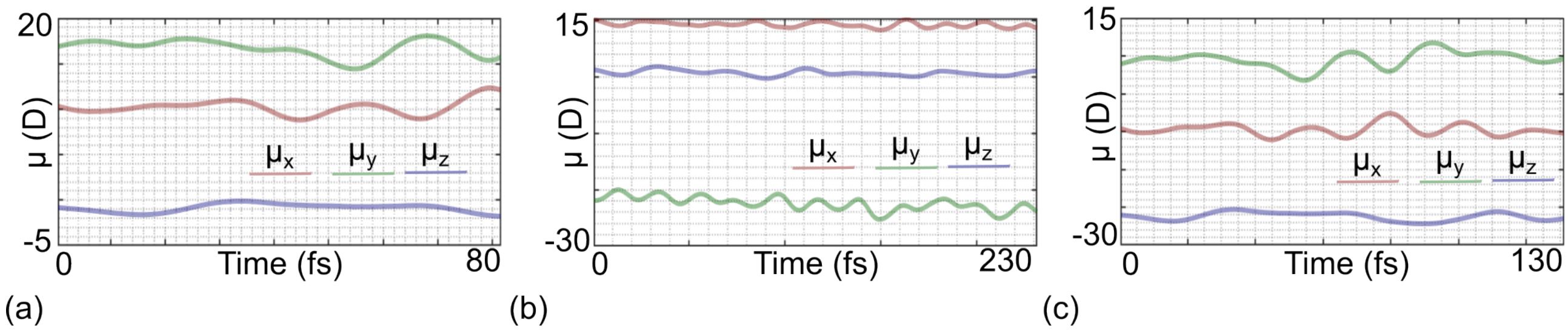


Figure S19: Time evolution of the molecular dipole components obtained from AIMD simulations for molecule M28 at $E_{\mathrm{ck}} = 0$ V/nm under three input-bias conditions: (a) $V_{\mathrm{in}} = 0.0$ V, (b) $V_{\mathrm{in}} = -0.32$ V, and (c) $V_{\mathrm{in}} = 0.32$ V. The AIMD time-averaged dipoles are more closely reproduced by the lowest-energy-conformer VACT than by the Boltzmann-weighted representation, highlighting the molecule-dependent character of the appropriate VACT selection.

## 10. Information Propagation Test

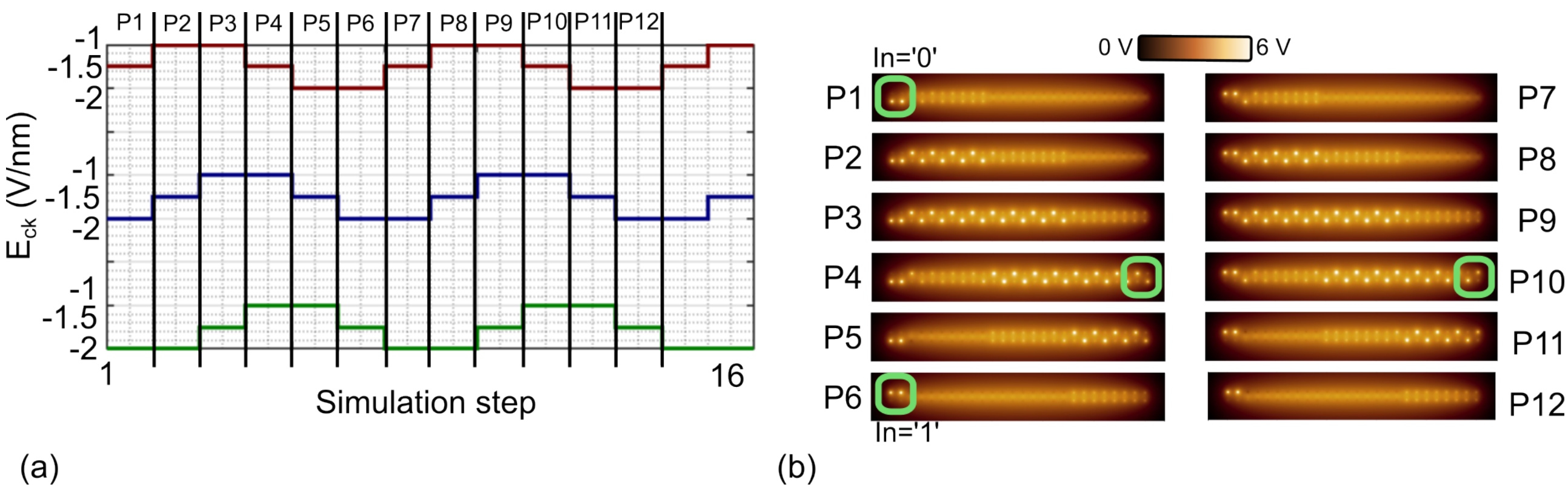


Figure S20: SCERPA information-propagation analysis of the M6 molecular wire. (a) Clock-field sequence applied to the three successive clocking zones during the simulation, with the dashed lines identifying the propagation snapshots shown in panel (b). (b) Electrostatic-potential maps corresponding to the successive propagation stages for logical inputs “0” and “1”, illustrating the evolution of the molecular polarization and the transmission of the logic state along the molecular wire using the validated VACT representation.
